# Anti-Inflammatory and Cytotoxic Compounds Isolated from Plants of *Euphorbia* Genus

**DOI:** 10.3390/molecules29051083

**Published:** 2024-02-29

**Authors:** Sarai Rojas-Jiménez, María Guadalupe Valladares-Cisneros, David Osvaldo Salinas-Sánchez, Julia Pérez-Ramos, Leonor Sánchez-Pérez, Salud Pérez-Gutiérrez, Nimsi Campos-Xolalpa

**Affiliations:** 1 Doctorado en Ciencias Biológicas y de la Salud, Universidad Autónoma Metropolitana-Xochimilco, Calzada del Hueso 1100, Ciudad de México 04960, Mexico; sarai_168@hotmail.com; 2 Facultad de Ciencias Químicas e Ingeniería, Universidad Autónoma del Estado de Morelos, Av. Universidad 1001, Col. Chamilpa, Cuernavaca 62209, Morelos, Mexico; mg.valladares@uaem.mx; 3 Centro de Investigación en Biodiversidad y Conservación, Universidad Autónoma del Estado de Morelos, Av. Universidad 1001, Col. Chamilpa, Cuernavaca 62209, Morelos, Mexico; davidos@uaem.mx; 4 Departamento de Sistemas Biológicos, Universidad Autónoma Metropolitana-Xochimilco, Calzada del Hueso 1100, Ciudad de México 04960, Mexico; jperez@correo.xoc.uam.mx; 5 Departamento de Atención a la Salud, Universidad Autónoma Metropolitana-Xochimilco, Calzada del Hueso 1100, Ciudad de México 04960, Mexico; tlsperez@correo.xoc.uam.mx

**Keywords:** *Euphorbia* genus, anti-inflammatory, anti-cancer activities

## Abstract

*Euphorbia* is a large genus of the Euphorbiaceae family. Around 250 species of the *Euphorbia* genus have been studied chemically and pharmacologically; different compounds have been isolated from these species, especially diterpenes and triterpenes. Several reports show that several species have anti-inflammatory activity, which can be attributed to the presence of diterpenes, such as abietanes, ingenanes, and lathyranes. In addition, it was found that some diterpenes isolated from different *Euphorbia* species have anti-cancer activity. In this review, we included compounds isolated from species of the *Euphorbia* genus with anti-inflammatory or cytotoxic effects published from 2018 to September 2023. The databases used for this review were Science Direct, Scopus, PubMed, Springer, and Google Scholar, using the keywords *Euphorbia* with anti-inflammatory or cytotoxic activity. In this review, 68 studies were collected and analyzed regarding the anti-inflammatory and anti-cancer activities of 264 compounds obtained from 36 species of the *Euphorbia* genus. The compounds included in this review are terpenes (95%), of which 68% are diterpenes, especially of the types ingenanes, abietanes, and triterpenes (approximately 15%).

## 1. Introduction

Inflammation is a homeostatic defense of the body against any injurious stimulus, whether physical, chemical, or biological [1]. It is characterized by the presence of pain, redness, swelling, heat, and loss of function, and it can be classified as acute or chronic. Acute inflammation is a protective response that disappears within minutes, hours, or a few days after the stimulus or injury. It is characterized by the release of phagocytes and mediators that act on endothelial cells, causing changes in vascular permeability and generating the migration of leukocytes and plasma proteins to produce edema. At this level, a generalized systemic reaction is triggered, and it is dynamic to resolve the inflammation. If unresolved, there is a risk that the inflammation could become chronic [2].

Chronic inflammation is long-term, lasting months to years, and it is characterized by the infiltration of macrophages, lymphocytes, and plasma cells into the injured tissue. It is a proliferation of fibroblasts and small blood vessels [2] producing pro-inflammatory cytokines, such as tumor necrosis factor-*α* (TNF-*α*), interleukin 6 (IL-6), and IL-8, and they stimulate reactive oxygen species (ROS), which are involved in modulating inflammation and activating the transcription factor NF-κβ [3].

Currently, in the treatment of inflammatory problems, steroidal (SAIDs) and non-steroidal anti-inflammatory drugs (NSAIDs) and disease-modifying antirheumatic drugs (DMARDs) are used. However, their constant or long-term use produces undesirable side effects on the renal, liver, gastric, cardiovascular, and central nervous systems [4].

The progress and permanence of inflammation are the reasons for most chronic diseases, and inflammation presents one of the major threats to the health and longevity of persons. Chronic inflammation is involved in several diseases, including, for example, Alzheimer’s, type 2 diabetes, obesity, hypertension, and cancer [5]. 

Cancer is a disease where some cells of the body grow uncontrollably and can blowout to other organs of the body; this disease is caused by mutations, and the inflammation process produces oxidative stress, which causes damage to DNA and initiates signaling pathways, thus deregulating the cell cycle and increasing the risk of developing cancer [6]. The most common treatment for cancer is chemotherapy, which produces side effects and can result in resistance to the compounds used [7].

Since ancient times, many cultures have used plants for therapeutic purposes as an important source of natural products for treating different health problems, such as inflammation and cancer. Recently, the research on medicinal plants has been increasing [8]; about 80% of chemotherapeutic drugs have been obtained from plants in addition to anti-inflammatory compounds [9].

### Ethnobotany

The Euphorbiaceae family is one of the most diverse families of flowering plants of angiosperms. This family contains around 6745 species in 317 genera, distributed mainly in the tropics and subtropics of the world [10]. In Mexico, *Euphorbia* species are found mainly in Nayarit, Veracruz, Chiapas, Michoacán, Oaxaca, Jalisco, Guerrero, Puebla, Sonora, Sinaloa, and Tamaulipas. Only about 250 species of the *Euphorbia* genus have been studied chemically and pharmacologically [11,12]; from these species, terpenes, flavonoids, alkaloids, coumarins, cyanogenetic glycosides, and mainly tannins have been isolated. Several reports show that some species have anti-inflammatory activity, which can be attributed to the presence of diterpenes, such as tiglians, ingenanes, and dafnanes. In addition, it was found that some diterpenes isolated from different *Euphorbia* species have anti-inflammatory and cytotoxic activity against some types of cancer [13,14,15].

The aim of this review is to provide an overview of scientific studies on 264 natural products isolated from 36 species of the *Euphorbia* genus with anti-inflammatory and cytotoxic activities reported from 2018 to September 2023. In Table 1 are shown the different species evaluated in this review.

In Table 2 is shown the anti-inflammatory activity of the compounds obtained from 16 species of *Euphorbia*.

In Figure 1, Figure 2, Figure 3, Figure 4, Figure 5, Figure 6, Figure 7, Figure 8, Figure 9 and Figure 10 are shown the structures of the compounds that evaluated their anti-inflammatory activity.

In Table 3 is shown the anti-cancer activity of the compounds obtained from 27 species of *Euphorbia*.

In Figure 11, Figure 12, Figure 13, Figure 14, Figure 15, Figure 16, Figure 17, Figure 18 and Figure 19 are shown the structures of the compounds that evaluated their cytotoxic activity. 

## 2. Discussion

At present, the study of natural products obtained from medicinal plants continues to be of great interest because they provide a wide range of compounds with pharmacological activity against diseases, such as cancer, diabetes, and cardiovascular and chronic respiratory diseases, which, according to the World Health Organization (WHO), are the leading causes of mortality worldwide [84]. Furthermore, these diseases involve acute and chronic inflammatory processes. For this reason, it is of great importance to conduct reviews of scientific studies that provide an overview of the molecules isolated from plants used in traditional medicine, such as those of the *Euphorbia* genus. In this review, 68 studies were collected and analyzed regarding the anti-cancer and anti-inflammatory effects of 264 compounds isolated from 36 species of the *Euphorbia* genus. The anti-inflammatory activity of 104 compounds was evaluated for NO inhibition on macrophages or BV-2-cells stimulated with LPS using the Griess assay. Also, we found that compounds 97–107 have been investigated through vivo studies on ear edema in mice induced with TPA or paw edema induced with carrageenan or histamine. The cytotoxic activity of 147 secondary metabolites was evaluated against human cancer cell lines. Both activities, anti-inflammatory and cytotoxic effects, were evaluated only in 14 metabolites isolated from *E. kansuensis* and *E. alatavica* (49), *E. kansui* (50), *E. lathyris* (68, 69, 74, 80, 83, 87), *E. maculate* and *E. pedroi* (95), *E. nerifolia* (116, 117, 118, 119), and *E. wallichii* and *E. fisheriana* (136).

Some species of the genus *Euphorbia* produce latex, also known as “milky sap.” These latexes are characterized by containing a variety of compounds with pharmacological activities [85]. In Table 1 is shown that the latexes obtained from *E. resinifera* and *E. umbellata* were extracted with methanol and a solution of 1% H_2_SO_4_, respectively. From the methanol extract of *E. resinifera,* latexes were isolated Euphatexols C (126), Euphatexols D (127), Euphatexols E (128), Euphatexols F (129), and Euphatexols G (130); all of them had anti-inflammatory activity (Table 2) [72]. From the latex of *E. umbellata* was obtained Euphol (206); its cytotoxic activity was evaluated on the K-562 and HL-70 cancer cell lines (Table 3) [81].

The compounds included in this review are terpenes (95%), of which 159 are diterpenes, especially abietanes and lathyranes; also, other diterpenes classes have been isolated from plants of the *Euphorbia* genus, such as labdanes (1–3, 255–258), abietanes (35, 36, 49, 136, 149–155,158–161, 166–171,173–190, 195–197, 220, 221, 247, 253, 254), lathyranes (9–11, 68–89, 230, 234, 259), jatrophanes (120–123, 204, 211–219, 228, 229, 262), rosanes (15–21, 138), atisanes (7, 8), kauranes (137, 172, 191–194, 248), beyeranes (4–6), ingenanes (24, 25, 57–67, 241–243, 245, 263), daphnanes (162–165), tiglianes (26, 37, 48, 156, 157), premyrsinanes (198–201), and ingols (12–14, 244, 246).

**Figure 20:**
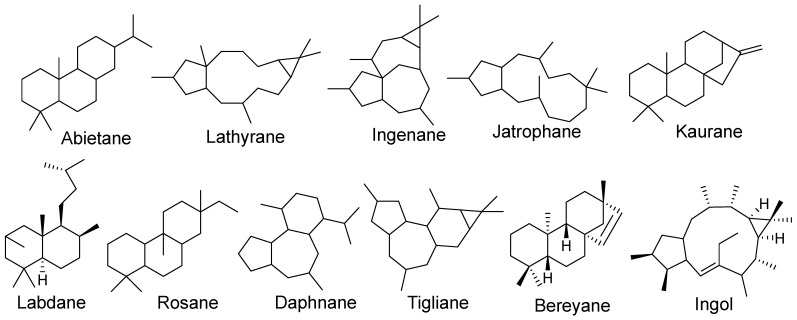
Hidrocarbon skeleton of *Euphorbia* diterpene classes.

Abietanes, rosanes, atisanes, beyeranes, and kauranes are characterized by three fused rings of six members, and some carbons are substituted with carbonyl or hydroxyl groups (264). Frequently, an olefin bond is found in the structure (Figure 20) [86].

Tiglianes, daphnanes, and ingenanes are characterized by a tetracyclic fused ring. Tiglianes usually have a configuration trans of the fusion of rings A and B and cis for the fusion of rings B and C. Daphnane diterpenoids have a tricyclic skeleton and the fusion of the rings A and B and B and C is trans [86]. Ingenanes diterpenes belong to the polycyclic diterpenoids related to daphnanes and tiglianes [87]; these diterpenes frequently contain hydroxyl and carbonyl groups and double bonds.

Lathyranes, jathropanes, and ingol are macrolides. Lathyranes diterpenes have a fused trycyclic system (5/11/3 members). Jathropanes have a bycyclo [9.3.0] pentadecane skeleton without a ring of cyclopropane. Ingol diterpenes are a subgroup of lathyranes characterized by a 5/11/3 carbon ring system with a 4,15-epoxy ring [88]. Their structure can contain hydroxyl, carbonyl, and ester groups and an olefin bond.

Labdanes are byciclic diterpenes with a branched six-carbon side chain [89]. Premyrsinanes are diterpenes with a [5-7-6-3] tetracyclic ring system [90].

These types of diterpenes show several pharmacological activities, some of which might be used clinically to treat health problems, such as cancer and inflammation [91]. 

Different researchers have found many diterpenes have anti-inflammatory activity through the inhibition of NF-κβ activation [86]; also, they diminish in macrophages stimulated with LPS, the production of TNF-α, NO, PGE2, the expression of COX-2, and iNOS mRNA [14]. 

For example, the factors L3 and L9 diminished the production of NO in LPS-stimulated macrophages by 61.85% and 63.68%, respectively. Also, both compounds had cytotoxic activity against BK (IC_50_ values of 7.9 and 6.1 µM, respectively) and BK-VIN (IC_50_ values of 8 and 5.7 µM, respectively) [58]. The compounds 1, 2, 70, and 137 promoted the suppression of iNOS expression and consequently decreased inflammation [17,54,83]. iNOS is the enzyme primarily responsible for the release of NO in inflammatory processes.

In another study, it was determined that the compounds Bisfischoid A and B (27, 28) isolated from *E. fischeriana* inhibited the activity of the soluble enzyme epoxide hydrolase (sEH) [30], and the compounds 29–34 obtained from *E. formosana* inhibited azurophilic degranulation of neutrophils [34]. On the other hand, compounds 70, 122, 123, and 137 diminished the levels of pro-inflammatory cytokines IL-1α, IL-6, and TNF-α [54,70,83]. The compounds 70 and 137 also inhibited the activation of COX-2 [54,83].

The compounds 18, 57, 61, and 69 suppressed NF-κβ, which is a light polypeptide gene enhancer in B cells produced and expressed by macrophages stimulated with LPS [53,54,55,60]; it promotes vasodilation and vascular permeability of blood vessels, facilitating the formation of edema and the recruitment of inflammatory cells around an injury [92]. For this reason, the compounds that decreased the levels of this polypeptide are candidates to be used in the treatment of inflammation. 

Cynsaccatol L (50) isolated from *E. lathyris* shows the highest effect on the inhibition of the production of NO for macrophages stimulated with LPS. This compound regulated the levels of TNF-α and IFN-ɤ and promoted the phagocytosis of macrophages of the M2 subtype [46] 

Cancer is a multifaceted ailment arising from mutations in cell proliferation. Interestingly, chronic inflammation has also been identified as a potential precursor to cancer in certain instances. The onset of cancer-promoting inflammation often precedes the formation of tumors. Notable examples of this connection can be found in certain conditions, such as *Helicobacter*-induced gastritis, chronic hepatitis, inflammatory bowel disease, and schistosomiasis-induced bladder inflammation. These conditions elevate the risk of developing several types of cancer, including, for example, colorectal, liver, stomach, and bladder cancer [93].

Many *Euphorbia* species contain compounds with cytotoxic activity. The mechanism of action of several types of diterpenoids has been investigated, and the results show that these compounds could have cytotoxic activity via induction of apoptosis through the suppression of IL-6-induced and STAT3 activation, the inhibition of topoisomerase II, and the impedance of NF-κβ activation [86].

The cytotoxic activity was evaluated mainly in the following cell lines: HepG2, MCF-7, C4-2B, CA2B/ENZR, A549, HL-60, HeLa, and more. Table 3 shows that the best cytotoxic effect on an MTT assay was obtained with 142–144 from *E. dendroides* on Huh-7, 156–159, 163, 173, 174, and 176 from *E. fischeriana* on HeLa, C4-2B, and CA-2B/ENZR, 210 from *E. grantii* on MCF7 and MCF7/ADR, 226 from *E. kansuensis* on RKO and MDA-MB-231, 230–231 from *E kansui* on GSC3, 242–243, 245, and 248 from *E. neriifolia* on A549, HL-60, and HepG2, 253 and 259 from *E. pekinensis* on K-562 and U-937, and 206 from *E. tirucalli* on DLD1, LNCaP, 5637, KYSE30, KYSE410, and P5N-1. Also, 136 isolated from several *Euphorbia* species demonstrated cytotoxic activity against HL-60, SMHC-7721, C4-2B, and C4-2B/ENZR. 

The compounds factor L1 and Euphosorophane I were evaluated with tests other than cytotoxicity in cancer cell lines [51,75]. Euphosorophane I (262) inhibited the function of transmembrane P-glycoprotein (P-gp), which has the function of an energy-dependent “drug pump.” Its overexpression promotes multidrug resistance (MDR). This effect was tested on drug-resistant MCF-7/ADR cells; it was found that compound 262 exhibited a P-gp-mediated MDR reversal [75].

The anti-cancer activity of factor L1 was studied in in vivo and in vitro models. This molecule presented cytotoxic and antitumor activity downregulating DDR1 in the tumor of SHI mice. This compound avoids anti-liver metastasis. Factor L1, Euphylbenzoate, and Glutinol induced cell death through apoptosis [39,51,73].

Factor L2 had a potent cytotoxic activity on A549 and induced apoptosis via the mitochondrial pathway, promoting the release of cytochrome C and the activation of caspase 3 and 9 [94]

## 3. Methods

The literature search of documents and reviews on the anti-inflammatory and cytotoxic studies of the different species of *Euphorbia* was conducted in the PubMed, Springer, Science Direct, and Google Scholar online databases. The recovered information that is presented was published in the last 5 years. Only studies on isolated compounds were considered. Different in vivo models were used to establish anti-inflammatory activity. With respect to the cytotoxic activity, different in vitro colorimetric methods were used, as well as different cancer cell lines (murine, human, and resistant). Table 1 shows the species, the collection place, the part of the plant, and the bioactive extract studied to isolate the active compounds.

## 4. Conclusions

In summary, plants of the *Euphorbia* genus are a source of compounds with anti-inflammatory and anti-cancer activities. Furthermore, different compounds shown in this review might lead to possible new therapies for inflammation and cancer to increase the options for the treatment of inflammatory diseases that afflict the world. Thirty-six species of *Euphorbia* were studied, and the specie that predominated was *E. lathyris*, which was researched in ten studies. 

One hundred forty-one compounds included in this review have anti-inflammatory activity; one hundred forty-three natural products have anti-cancer effects; and ten molecules present both activities. 

This review shows that 159 diterpenes were isolated from the *Euphorbia* genus, including 55 abietanes, 27 lathyranes, 17 ingenanes, 16 jathropanes, 8 rosanes, 7 kauranes, 7 labdanes, 5 tiglianes, 5 permyrsinanes, 4 daphnanes, 3 beyeranes, 2 atisanes, and 3 others.

Cynsaccatol (50) isolated from *E. lathyris* shows the greatest effect on the inhibition of the production of NO for macrophages stimulated with LPS. (4*R*,5*S*,8*S*,9*R*,10*S*,13*R*,16*S*)-ent-16α,17-dihydroxy-19-tigloyloxykauran-3-one (248) and Euphorbia factors L1 and L3 have good cytotoxic activity. These results show that the compounds 50, 68, 69, and 248 are promising to develop new drugs.

## Figures and Tables

**Figure 1:**
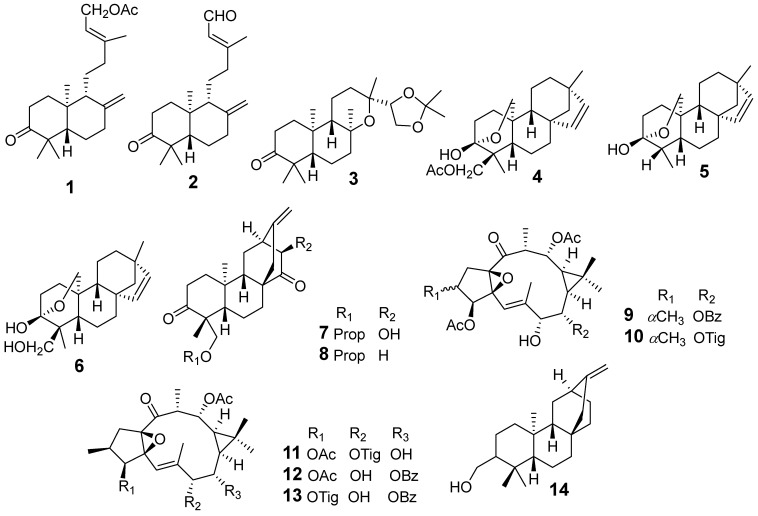
Structures of compounds isolated from *E. antiquorum*.

**Figure 2:**
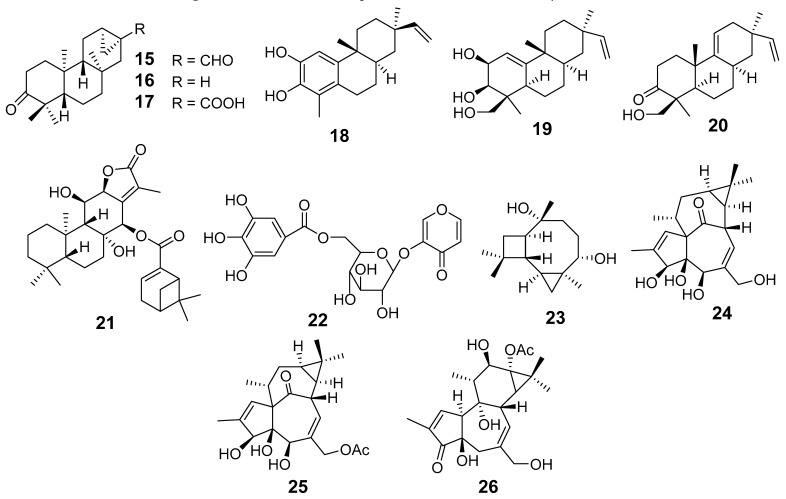
Structures of compounds isolated from *E. atoto* and *E. ebracteolata*.

**Figure 3:**
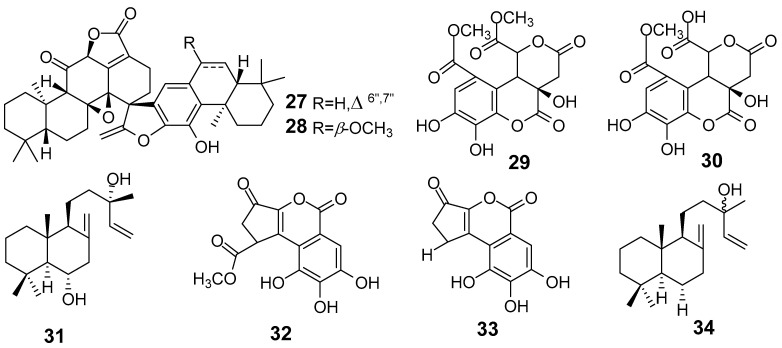
Structures of compounds isolated from *E. fischeriana* and *E. formasana*.

**Figure 4:**
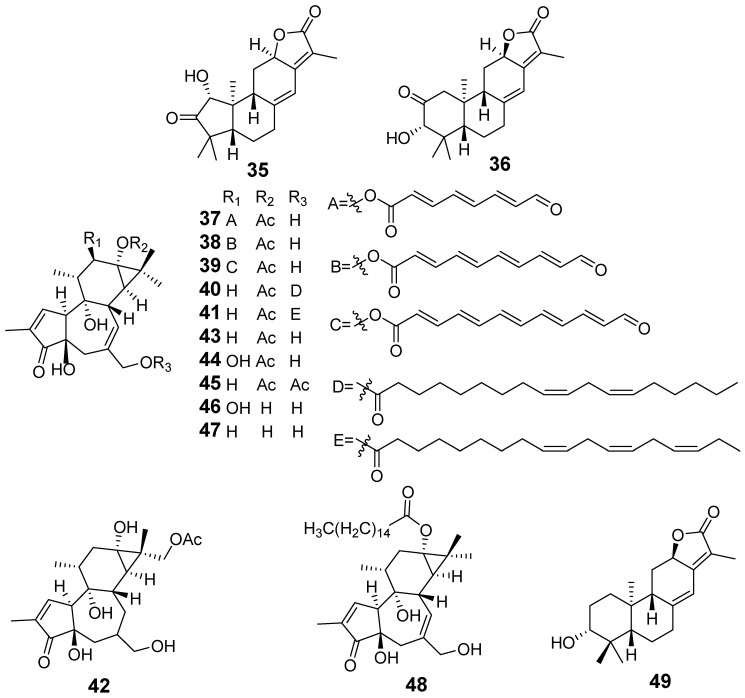
Structures of compounds isolated from *E. helioscopia* and *E. kansuensis*.

**Figure 5:**
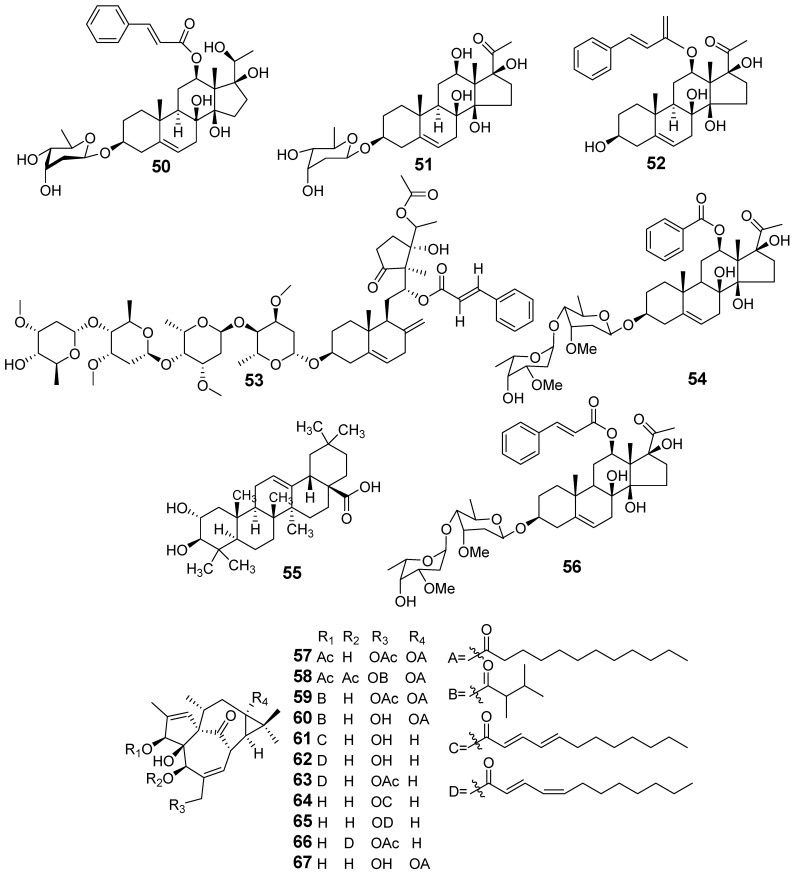
Structures of compounds isolated from *E. kansui*.

**Figure 6:**
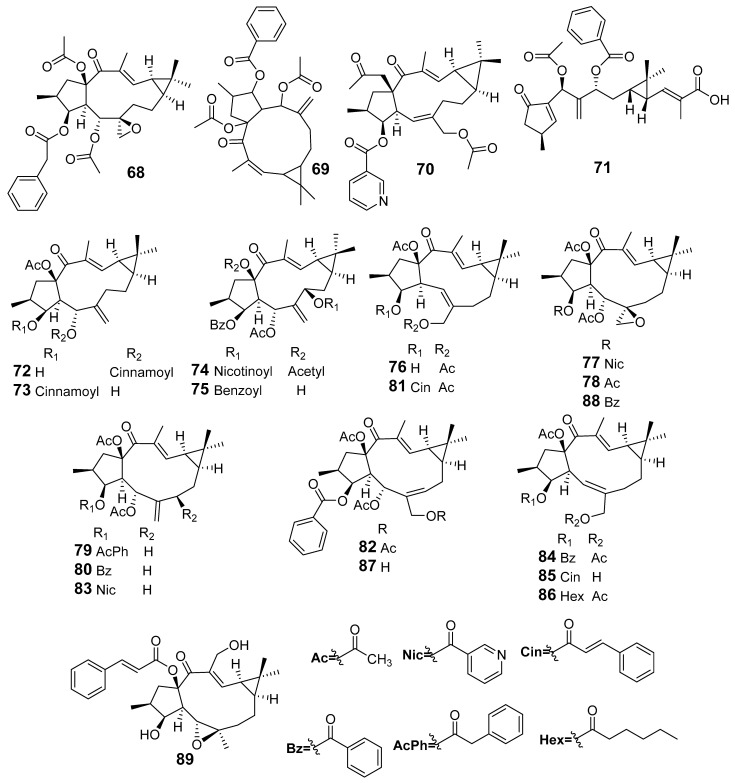
Structures of compounds isolated from *E. lathyris*.

**Figure 7:**
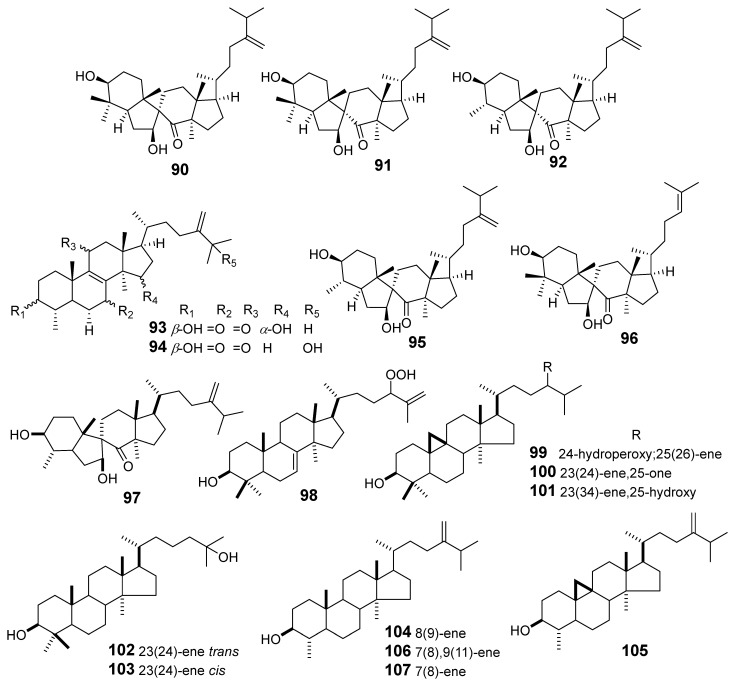
Structures of compounds isolated from *E. maculata*.

**Figure 8:**
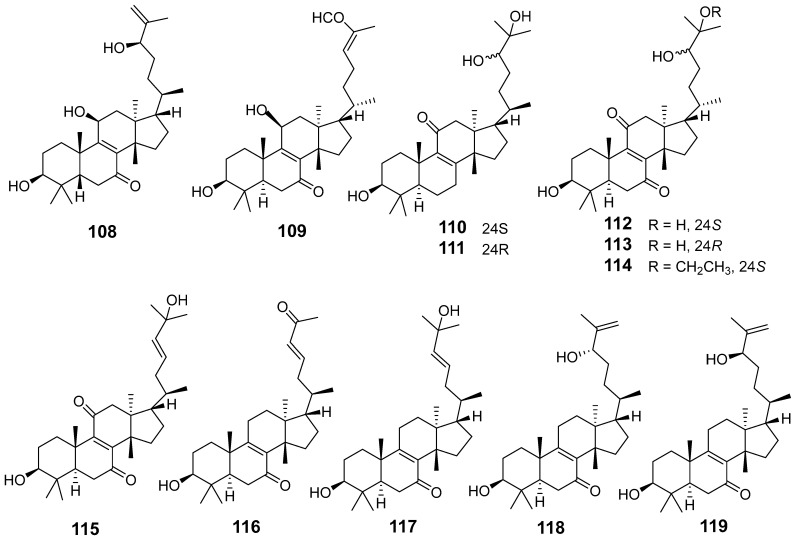
Structures of compounds isolated from *E. neriifolia*.

**Figure 9:**
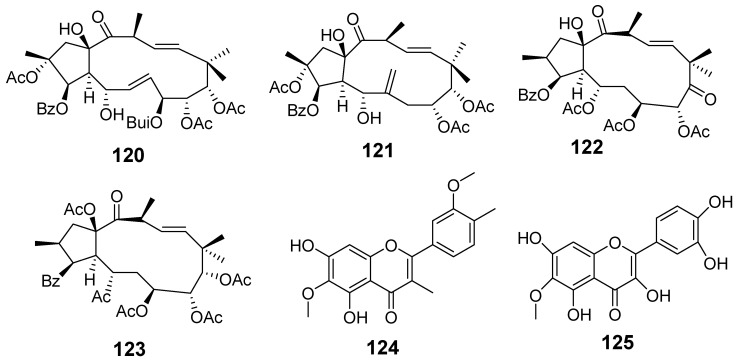
Structures of compounds isolated from *E. peplus* and *E. pulcherrima*.

**Figure 10:**
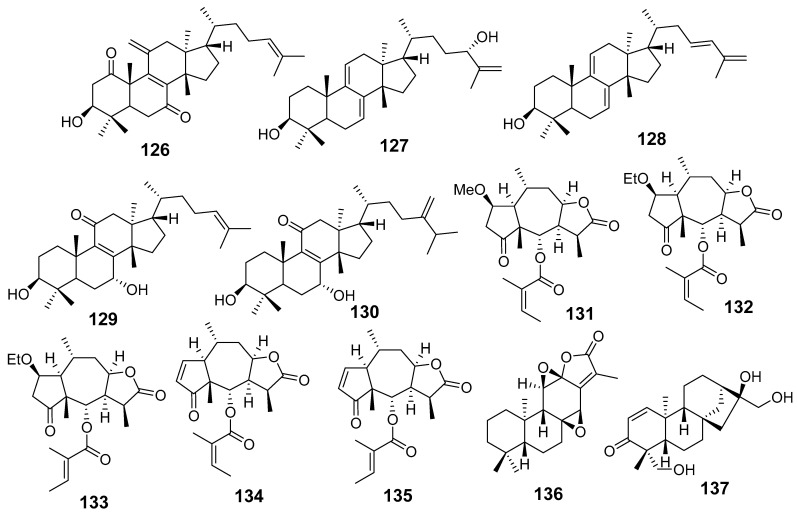
Structures of compounds isolated from *E. resinifera*, *E. thymifolia,* and *E. wallichii*.

**Figure 11:**
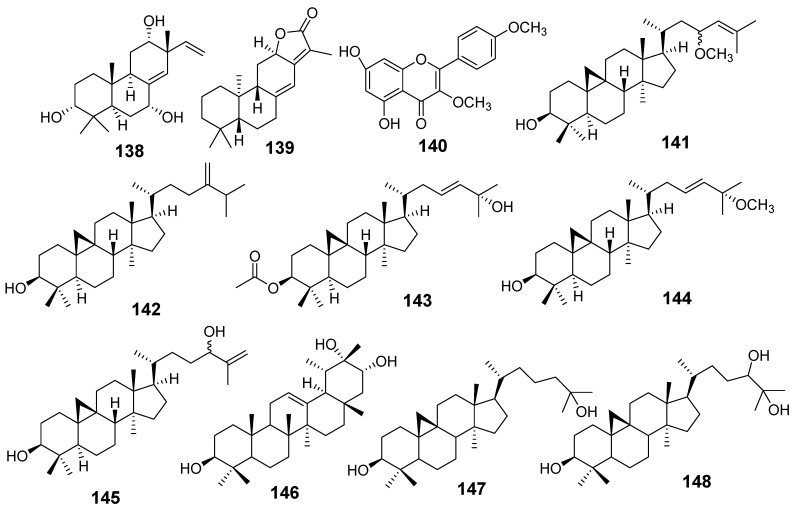
Structures of compounds isolated from *E. alatavica*, *E. balsamifera*, *E. dendroides*, and *E. denticulata*.

**Figure 12:**
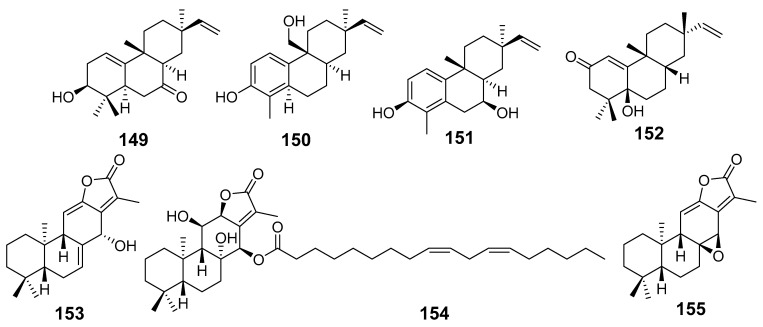
Structures of compounds isolated from *E. ebracteolata*.

**Figure 13:**
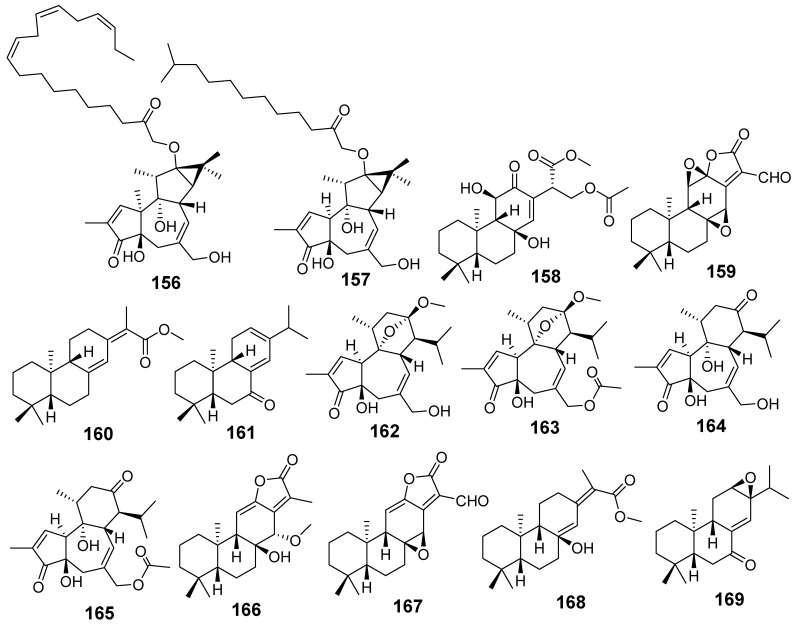

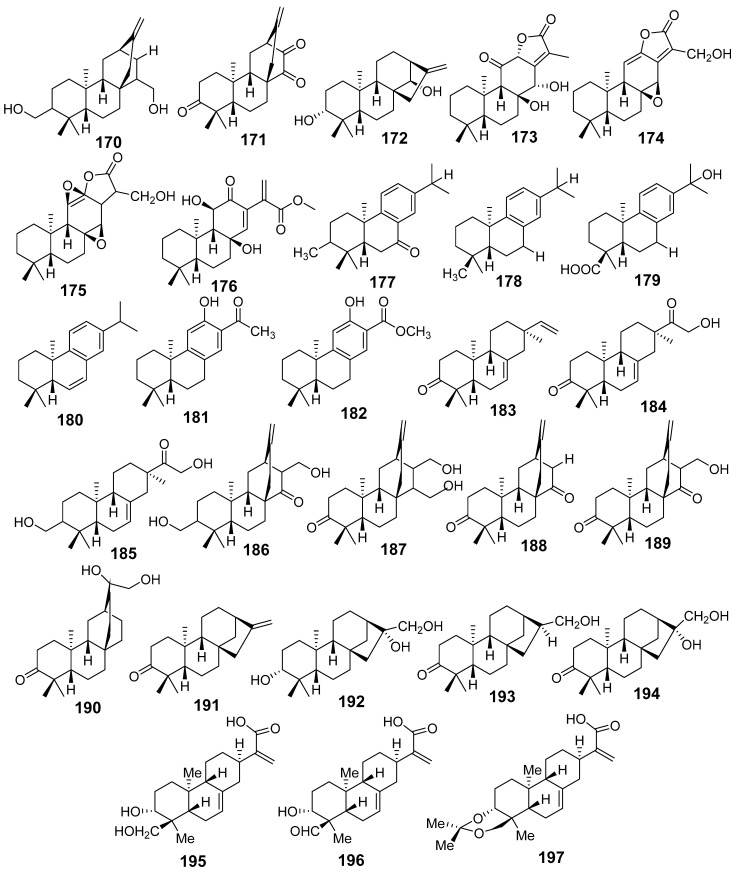
Structures of compounds isolated from *E. fisheriana*.

**Figure 14:**
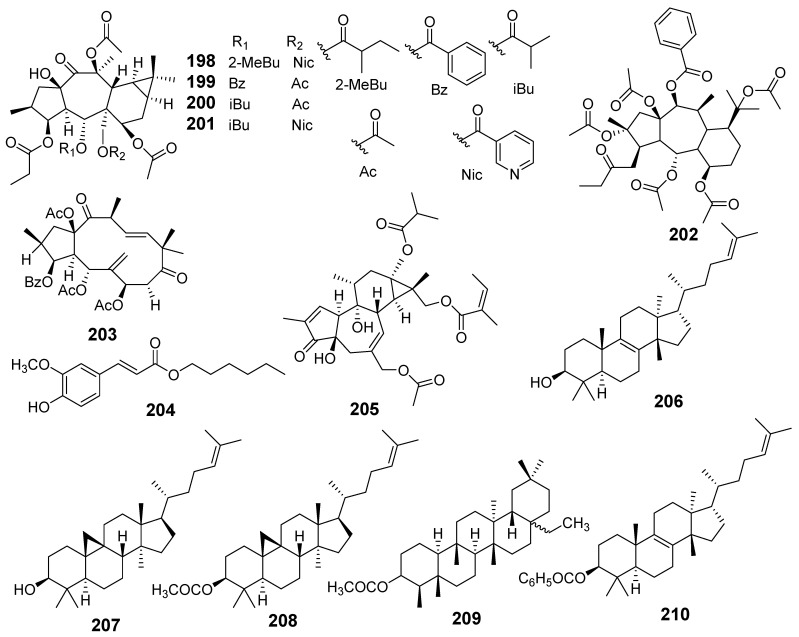
Structures of compounds isolated from *E. gedrosiaca*, *E. glomerulans*, *E. grandicornis*, and *E. grantii*.

**Figure 15:**
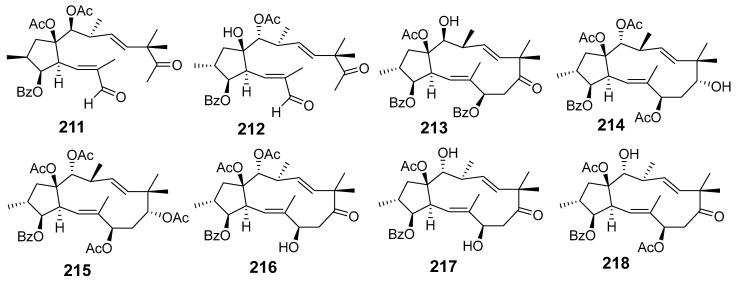

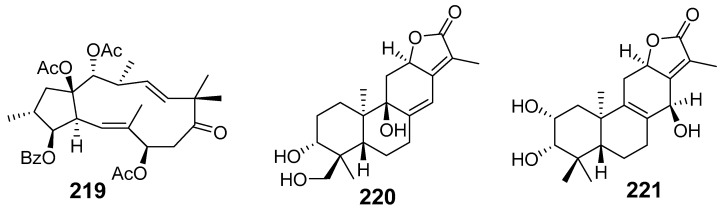
Structures of compounds isolated from *E. helioscopia*.

**Figure 16:**
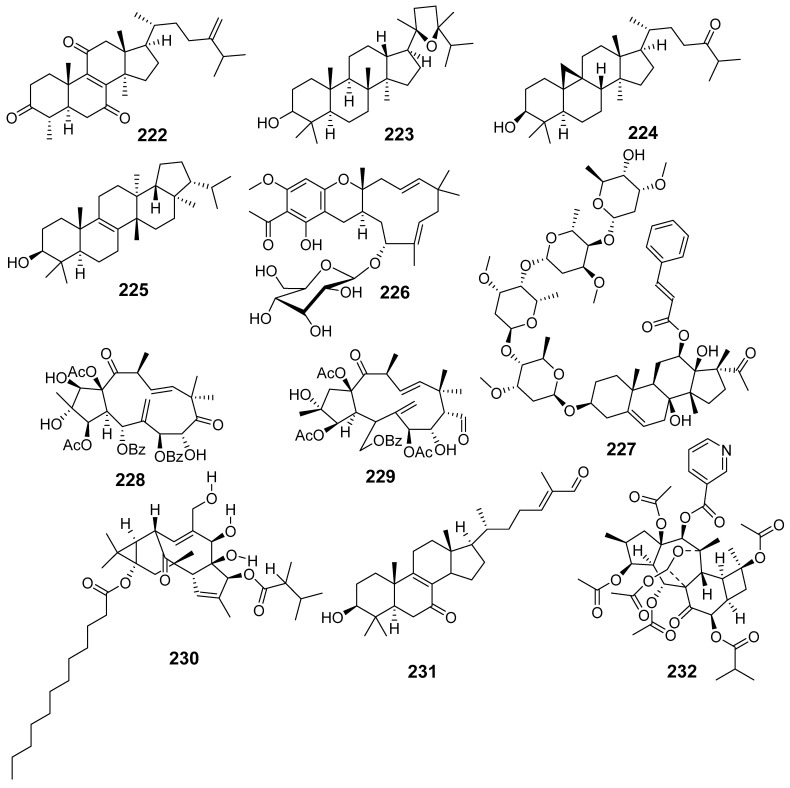
Structures of compounds from *E. hypericifolia*, *E. kansuensis*, *E. kansui*, and *E. kopetdaghi*.

**Figure 17:**
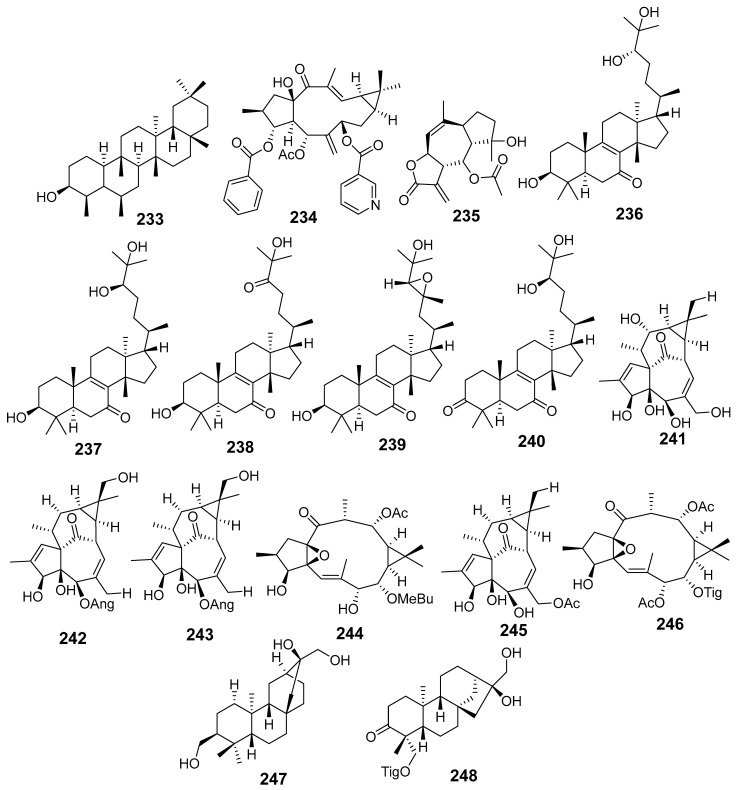
Structures of compounds isolated from *E. lactea*, *E. lathyris*, *E. microsphaera*, and *E. neriifolia*.

**Figure 18:**
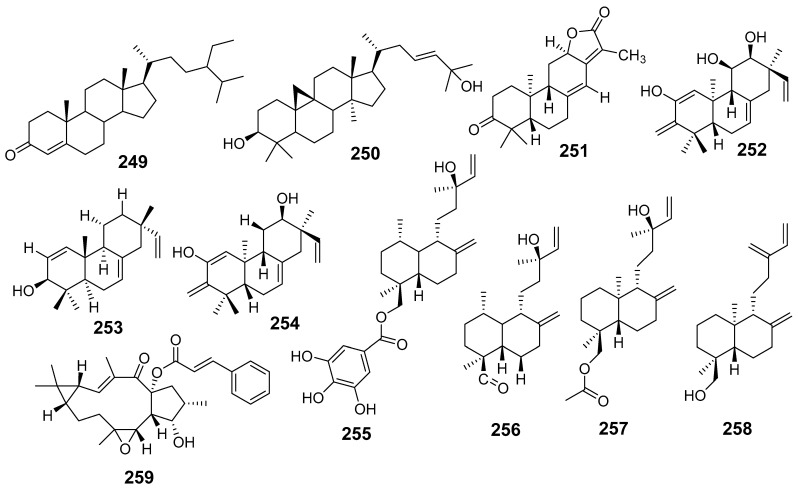
Structures of compounds isolated from *E. pedroi* and *E. pekinensis*.

**Figure 19:**
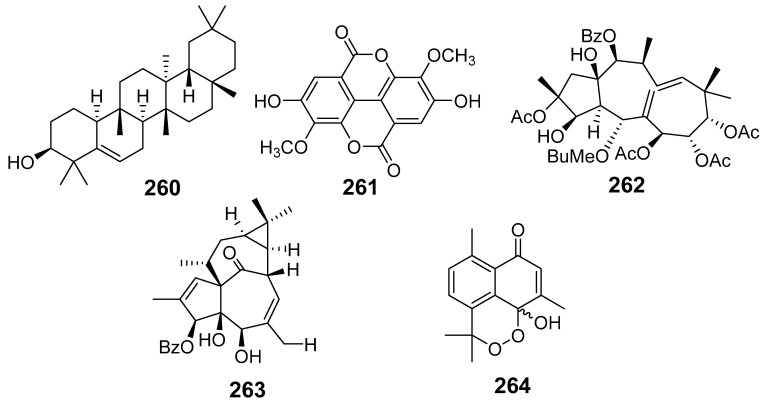
Structures of compounds isolated from *E. saudiarabica*, *E. schimperiana*, *E. sororia*, *E. stracheyi*, and *E. tirucalli*.

**Table 1:** Species of *Euphorbia* analyzed in this review, 2018–2023.

Species	Collection Place	Plant Material	Extract Solvent
*E. alatavica* [16]	China	Stems	Acetone
*E. antiquorum* [17,18]	Thailand	Aerials parts	Methanol
	China	Stems	Methanol
*E. atoto* [19]	China	Aerial parts	Ethanol
*E. balsamifera* [20]	Saudi Arabia	Aerial parts	Ethanol
*E. dendroides* [21]	Egypt	Aerial parts	Methanol
*E. denticulata* [22]	Iran	Whole plant	Acetone
*E. ebracteolata* [23,24,25,26,27]	China	Roots	Ethanol
	Korea		Methanol
	China		Ethanol
*E. fischeriana* [28,29,30,31,32,33]	Mongolia	Roots	Ethanol
	China		Acetone
*E. formosana* [34]	Taiwan	Roots	Methanol
*E. gedrosiaca* [35]	Iran	Aerial parts	Dichloromethane: Acetone
*E. glomerulans* [36]	China	Whole plant	Acetone
*E. grandicornis* [37,38]	South Africa	Aerial parts and roots	Dichloromethane
	Hungary	Aerial parts	Methanol
*E. grantii* [39]	Egypt	Aerial parts	Methanol
*E. helioscopia* [40,41,42]	China	Whole plant	Ethanol
			Methanol
		Aerials parts	Ethanol
*E. hypericifolia* [43]	China	Aerial parts	Ethanol
*E. kansuensis* [44,45]	China	Roots	Ethanol
*E. kansui* [46,47,48]	China	Roots	Ethanol
*E. kopetdaghi* [49]	Iran	Aerial parts	Dichloromethane: Acetone 2:1
*E. láctea* [50]	Thailand	Aerial parts	Ethanol
*E. lathyris* [51,52,53,54,55,56,57,58,59,60]	China	Seeds	Ethanol
			Petroleum Ether
			Ethanol
			Ethanol
			Petroleum Ether
			Ethanol
			Ethanol
			Ethanol
	South Korea	Seeds	Methanol
*E. maculata* [61,62]	China	Whole plant	Ethanol
	Japan	Whole plant	Methanol
*E. microsphaera* [63]	Iran	Aerial parts	Chloroform
*E. neriifolia* [64,65,66,67]	Taiwan	Stems	Ethanol
	China	Aerial parts	Ethanol
		Whole plant	Acetone: Water 3:1
*E. pedroi* [68]	Portugal	Aerial parts	Methanol
*E. pekinensis* [69]	China	Roots	Ethanol
*E. peplus* [70]	China	Leaves	Methanol
*E. pulcherrima* [71]	Pakistan	Whole plant	Methanol
*E. resinifera* [72]	China	Latex	Methanol
*E. saudiarabica* [73]	Saudi Arabia	Aerial parts	Methanol
*E. schimperiana* [74]	Saudi Arabia	Aerial parts	Ethanol
*E. sororia* [75]	China	Fructus	Ethanol
*E. stracheyi* [76]	China	Whole plant	Methanol
*E. thymifolia* [77]	China	Aerial parts	Ethanol
*E. tirucalli* [78,79,80]	Vietnam	Whole plant	Ethanol
	Brazil	Sap	Hexane
*E. umbellata* [81]	Brazil	Latex	H_2_SO_4_ 1%
*E. wallichii* [82,83]	China	Whole plant	Methanol

**Table 2:** The anti-inflammatory activity of the compounds obtained from 16 species of *Euphorbia*.

Species	Active Compounds	Biological Model	Results	Ref.
*E. antiquorum*	Ent-15-Acetoxylabda-8(17),13*E*-diene-3-one (1)	Griess assay J774.A1 cells stimulated LPS NO	IC_50_ (μM)11.7	[17]
	Ent-15-Oxolabda-8(17),13*E*-diene-3-one (2)		12.5	
	Ent-13-epi-8,13-epoxy-14α,15-isopropylidenedioxylabdane-3-one (3)		44.6	
	Ent-3β,20-Epoxy-3α-hydroxy-15-beyeren-18-acetate (4)		36.6	
	Ent-3β,20-epoxy-3α-hydroxy-18-norbeyer-15-ene (5)		40.4	
	Rhizophorin B (6)		16.1	
	Ent-15-Acetoxylabda-8(17),13*E*-diene-3-one (1)	Western blot iNOS	IC_50_ (μM) 11.7	
	Ent-15-Oxolabda-8(17),13*E*-diene-3-one (2)		12.5	
	Euphorin A (7)	Griess assay BV-2 cells stimulated LPS NO	IC_50_ (µM) 35.8	[18]
	Euphorin B (8)		41.4	
	Euphorin D (9)		32.0	
	Euphorin E (10)		40.7	
	3,12-*O*-diacetyl-7-*O*-[(*E*)-2-methyl-2-butenoyl]-8,12-diepjing-ol (11)		49.2	
	3,12-diacetyl-8-benzoylingol (12)		14.5	
	12-*O*-acetyl-8-*O*-benzoylingol-3-tiglate (13)		14.9	
	Ent-(3α,5β,8α,9β,10α,12α)-3-hydroxyatis-16-en-14-one (14)		31.6	
*E. atoto*	3-oxo-ent-trachyloban-17-oic acid (15)	Griess assay RAW264.7 cells stimulated LPS NO	IC_50_ (µM) 41.61	[19]
	Ent-kauran-16β-ol-3-one (16)		16.00	
	Ent-16-hydroxy-3-oxosanguinane (17)		33.41	
*E. ebracteolata*	Ebractenoid F (18)	Griess assay RAW264.7 cells stimulated LPS NO	IC_50_ (µg/mL) 2.39	[24]
		SEAP Assay NF-*k*B	Decreased NF-*k*B. Inhibited the phosphorylation of Akt and mitogen-activated protein kinases (MAPKs)	
		Western blot	Inhibited levels of IL-6 and IL1	
	Ebractenoid O (19)	Griess assay RAW264.7 cells stimulated LPS NO	IC_50_ (µM) 6.04	[27]
	Ebractenoid P (20)		10.23	
	Ebractenoid Q (21)		1.97	
	γ-pyrone-3-*O*-β-d-(6-galloyl)-glucopyranoside (22)		42.49	
	Tricyclohumuladiol (23)		13.21	
	Ingenol (24)		6.25	
	Ingenol-20-acetate (25)		6.73	
	Langduin A4 (26)		18.50	
*E. fischeriana*	Bisfischoid A (27)	Assay Inhibition of sEH	IC_50_ (µΜ) 9.90	[30]
	Bisfischoid B (28)		10.29	
*E. formosana*	Euphormin A (29)	Superoxide Anion In human neutrophils stimulated with formyl-L methionyl-l-leucyl-l-phenylalanine/cytochalasin B	IC_50_ (µM) 4.51	[34]
	Euphormin B (30)		3.68	
	Larixol (31)		3.81	
	Methylbrevifolincarboxylate (32)		0.68	
	Brevifolin (33)		1.39	
	Euphormins A (29)	Elastase Release In human neutrophils stimulated with formyl-L methionyl-l-leucyl-l-phenylalanine/cytochalasin B	IC_50_ (µM) >10	
	Euphormins B (30)		>10	
	Larixol (31)		>10	
	Methylbrevifolincarboxylate (32)		>10	
	Brevifolin (33)		>10	
	*epi*-manool (34)		8.07	
*E. helioscopia*	Euphohelide A (35)	Griess assay RAW264.7 cells stimulated LPS NO	IC_50_ (µM) 32.98	[40]
	Helioscopinolide C (36)		33.82	
*E. kansuensis*	Euphkanoid A (37)	Griess assay RAW264.7 cells stimulated LPS NO	IC_50_ (µM) 9.41	[44]
	Euphkanoid B (38)		11.3	
	Euphkanoid C (39)		5.92	
	Euphkanoid D (40)		24.5	
	Euphkanoid E (41)		35.3	
	Euphkanoid F (42)		4.8	
	Prostratin (43)		45.9	
	Phorbol-13-acetate (44)		44.8	
	12-deoxyphorbol-13,20-diacetate (45)		37.9	
	Phorbol (46)		47.0	
	12-deoxyphorbol (47)		35.7	
	12-deoxyphorbol-13-hexadecanoate (48)		24.3	
	Helioscopinolide A (49)		23.5	
*E. kansui*	Cynsaccatol L (50)	Na+-K+-ATPase Analysis	Induced inactivation of AKT and ERK due to the downregulation of ATP1A1 expression	[46,47]
	Cynotophylloside B (51)	Western blot	Inhibited the phosphorylation of AKT and mTOR, as well as upregulating the expression of LC3-Band p62	
	Cynsaccatol L (50)	Griess assay RAW264.7 cells stimulated LPS NO	IC_50_ (µM) 0.02	
	Cynotophylloside B (51)		9.10	
	Kidjolanin (52)		30.7	
	Wilfoside G (53)		1.77	
	Cynotophylloside J (54)		17.39	
	Maslinic acid (55)		17.38	
	Kidjoranin 3-*O*-α-diginopyranosyl-(1→4)-β- Cymaropyranoside (56)		2.79	
	Euphorkan A (57)	Griess assay RAW264.7 cells stimulated LPS NO	IC_50_ (µM) 4.90	[48]
	Euphorkan B (58)		10.4	
	3-*O*-(2,3-dimethylbutanoyl)-13-*O*-dodecanoyl-20-*O*-acetylingenol (59)		5.69	
	3-*O*-(2,3-dimethylbutyryl)-13-*O*-n-dodecanoyl-13-hydroxyingenol (60)		5.80	
	3-*O*-(2′*E*,4′*E*-decadienoyl) ingenol (61)		2.78	
	3-*O*-(2′*E*,4′*Z*-decadienoyl) ingenol (62)		10.6	
	3-*O*-(2′*E*,4′*Z*-decadienoyl)-20-*O*-acetylingenol (63)		2.86	
	20-*O*-(2′*E*,4′*E*-decadienoyl) ingenol (64)		9.05	
	20-*O*-(2′*E*,4′*Z*-decadienoyl) ingenol (65)		9.45	
	20-*O*-acetyl-[5-*O*-(2′*E*,4′*Z*)-decadienoyl]-ingenol (66)		4.60	
	13-*O*-docecanoylingenol (67)		8.86	
	Euphorkan A (57)	Luciferase assay NF-κB	IC_50_ (µM) 11.0	
	3-*O*-(2′*E*,4′*E*-decadienoyl) ingenol (61)		17.9	
*E. lathyris*	Euphorbia Factor L1 (68)	Cytokines were determined using ELISA	SHI-induced inflammatory cell infiltration and IL-1β, IL-6, TNF-α were decreased	[52]
		Western blot	Treatment with EFL1 downregulated DDR1 protein expression and immuno-reactivity in SHI mice, leading to the surge of CD4+, CD8+, and CD49b+ (NK) T cells	
	Euphorbia Factor L3 (69)	Fibroblast-like synoviocytes (FLSs)	Ameliorated inflammatory phenotype FLSs (decreased viability, migration, invasion, and cytokine production)	[53]
		Collagen-induced arthritis (CIA)	Inhibited arthritic progression	
		Wester blotting and immunofluorescence	Inhibited nuclear translocation of the p65	
		Molecular analysis	Target of EFL3 is RACI	
	Euplarisan A (70)	Griess assay RAW264.7 cells stimulated LPS NO	IC_50_ (μM) 7.50	[54]
		Enzyme-linked immunoassay (ELISA)	Inhibited IL-1β, IL-6, and TNF-α	
		Western blot assay	Decreased the expression of iNOS, COX-2, and p-IκBα	
	Lathyranoic acid A (71)	Griess assay BV-2 cells stimulated LPS NO	% Inhibitory 74.51	[55]
	Euphorbia Factor L3 (69)		61.85	
	Euphorbia Factor L31 (72)		50.46	
	Euphorbia Factor L30 (73)		50.01	
	Euphorbia Factor L9 (74)		63.68	
	Euphorbia Factor L11 (75)		76.66	
	Euphorbia Factor L3 (69)	Griess assay RAW264.7 cells stimulated LPS NO	IC_50_ (μM) 11.24	[56]
	Euphorbia Factor L29 (76)	Griess assay RAW264.7 cells stimulated LPS NO	IC_50_ (µM) 47.9	[60]
	Euphordracunculin C (77)		12.7	
	Epoxyboetirane A (78)		26.2	
	Euphorbia Factor L1 (68)		12.7	
	Deoxy Euphorbia Factor L1 (79)		47.0	
	Euphorbia Factor L2 (80)		16.2	
	Euphorbia Factor L3 (69)		15.0	
	Euphorbia Factor L7a (81)		44.4	
	Euphorbia Factor L7b (82)		23.9	
	Euphorbia Factor L8 (83)		30.3	
	Euphorbia Factor L9 (74)		11.2	
	Euphorbia Factor L17 (84)		48.5	
	Euphorbia Factor L22 (85)		16.6	
	Euphorbia Factor L23 (86)		19.5	
	Euphorbia Factor L24 (87)		18.2	
	Euphorbia Factor L25 (88)		28.9	
	Jolkinol A (89)		12.5	
*E. maculata*	Spiromaculatol A (90)	Griess assay RAW264.7 cells stimulated LPS NO	IC_50_ (μM) 23.1	[61]
	Spiromaculatol B (91)		17.4	
	Spiromaculatol C (92)		8.8	
	Euphomaculatoid B (93)		31.3	
	Euphomaculatoid D (94)		15.9	
	Spiropedroxodiol (95)		12.7	
	Spiroinonotsuoxodiol (96)		20.6	
	4-methyl-3,7-dihydroxy-7 (8 → 9) *abeo*-lanost-24 (28) -en-8-one (97)	Ear edema in induced mouse by TPA	ID_50_ (nM/ear) 803	[62]
	24-hydroperoxylanost-7,25-dien-3β-ol (98)		356.3	
	3-hydroxycycloart-25-*ene*-24-hydroperoxide (99)		301.7	
	3β-hydroxy-26-nor-9,19-cyclolanost-23-en-25-one (100)		558	
	Cicloart-23(24)-ene-3β,25-hydroxy (101)		355.7	
	(23*E*)-3,25-dihydroxythirucalla-7,23-diene (102)		855	
	(23*Z*)-3,25-dihydroxy-thyrucalla-7,23-diene (103)		1087	
	Obtusifoliol (104)		87.7	
	4α, 14α-dimethyl-5α-ergosta-7,9 (11), 24 (28) -trien-3β-ol (105)		363.1	
	Gramisterol (106)		204	
	Cycloeucalenol (107)		463.9	
*E. neriifolia*	Neritriterpenol H (108)	Griess assay RAW264.7 cells stimulated LPS	All compounds inhibited IL-6	[64]
	Neritriterpenol I (109)			
	Neritriterpenol J (110)			
	Neritriterpenol K (111)	ELISA kits	Secretion in a dose-dependent manner	
	Neritriterpenol L (112)			
	Neritriterpenol M (113)			
	Neritriterpenol N (114)			
	11-Oxo-kansenonol (115)			
	Sooneuphanone B (116)	Griess assay RAW264.7 cells stimulated LPS NO	% inhibition 20 (µg/mL) 58.4%	[65]
	(23*E*)-eupha- 8,23-diene-3β,25-diol-7-one (117)		27–39%	
	(+)-(24*S*)-eupha-8,25-diene-3β,24-diol-7-one (118)			
	(24*R*)-eupha-8,25-diene-3β,24-diol-7-one (119)			
*E. peplus*	Euphopepluanone N (120)	Griess assay RAW264.7 stimulated LPS	Inhibited NO production	[70]
	Euphopepluanone B (121)			
	(2S*, 3*S*, 4R*, 5R*, 7S*, 13R*, 15R*)−3, 5, 7,15-tetraacetoxy-9, 14-dioxojatropha-6(17), 11*E*-diene (122)			
	11*E*-diene-9, 14-dione (123)	RT-qPCR analysis	Inhibited generation of cytokines (Il-6, IL-1β, TNF-α)	
	(11*E*, 2*S*, 3*S*, 4*R*, 5*R*, 7*S*, 13*R*, 15*R*)−3, 5, 7,15-tetraacetoxy-9, 14-dioxojatropha-6(17), 11*E*-diene (122)			
*E. pulcherrima*	Spinacetin (124)	Paw edema induced by Carrageen	% Edema inhibition 79.22	[71]
	Patuletin (125)		89.01	
	Spinacetin (124)	Paw edema histamine model	78.33	
	Patuletin (125)		94.00	
*E. resinifera*	Euphatexols C (126)	Griess assay RAW264.7 cells stimulated LPS NO	IC_50_ (μM) 22.30	[72]
	Euphatexols D (127)		48.04	
	Euphatexols E (128)		21.89	
	Euphatexols F (129)		38.15	
	Euphatexols G (130)		41.15	
*E. thymifolia*	(1*S*, 2*R*, 5*R*, 6*S*, 7*R*, 8*R*, 10*R*, 11*S*)-4-oxo-2-methoxy-6-angeloyloxy-pesudoguai-8,12-olide (131)	Griess assay BV-2 stimulated LPS NO	IC_50_ (μM) 6.46	[77]
	Minimolide B (132)		15.32	
	4-oxo-2-ethoxy-6-tigloyloxy-pesudoguai-8,12-olide (133)		7.15	
	6-*O*-angeloylplenolin (134)		0.41	
	6-*O*-tigloyl-11,13-dihydrohelenalin (135)		0.54	
*E. wallichii*	Jolkinolide B (136)	Griess assay RAW264.7 stimulated LPS NO	IC_50_ (µM) 3.84	[82]
		ELISA assay IL-6 TNF-α	IC_50_ (µM) >4 >16	
	Wallkaurane A (137)	Griess assay RAW264.7 stimulated LPS NO	IC_50_ (µM) 3.84	[83]
		ELISA assay	The production of inflammatory cytokines (IL-6 and TNF-α)	
		Western blot	Increased the expression of the antiapoptotic marker Bcl-2. Decreased the expression of iNOS and COX-2	

**J774.A1** cells macrophages isolated from ascites of female mice with reticulum cell sarcoma; **RAW264.7** cells are a macrophage-like, Abelson leukemia virus-transformed cell line derived from BALB/c mice; **BV-2** cells are a unique type of microglial cells derived from C57/BL6 murine; **Griess** assay is a colorimetric method for the quantitative analysis of nitrites; **CCK-8 assay**: Cell Counting Kit-8 using WST-8 (2-(2-methoxy-4-nitrophenyl)-3-(4-nitrophenyl)-5-(2,4-disulfophenyl)-2*H*-tetrazolium, monosodium salt); **LPS:** Lipopolysaccharide or endotoxin is the major component of the outer membrane of Gram-negative bacteria; **DDR1:** Discoid in domain receptor 1; **TPA:** 12-O-Tetradecanoylphorbol-13-acetate; **NO:** nitric oxide; **IL-1β:** Proinflammatory cytokine 1β; **IL-6:** Proinflammatory cytokine 6; **TNF-α:** tumor necrosis factor α; **NF-kβ:** Nuclear Factor enhancer of kappa light chains of activated B cells; **iNOS:** Inducible Nitric Oxide Synthase; **SOD:** Superoxide Dismutase; **sEH**: Soluble Epoxide Hydrolase; **RT-qPCR:** Quantitative real-time PCR.

**Table 3:** The cytotoxic activity of the compounds obtained from 27 species of *Euphorbia*.

Species	Compounds	Biological Model	Result	Ref
*E. alatavica*	3α,7α,12α-trihydroxyisopimara-8(14), 15-diene (Alatavnol A) (138)	MTT assay MCF7 A549	IC_50_ (µg/mL) 14.327 12.033	[16]
	Helioscopinolide A (49)	HeLa MCF7	23.802 33.476	
	Jolkinolide E (139)	MCF7	22.066	
*E. balsamifera*	Kampferol-3,4′-dimethyl ether (140)	MTT assay HePG2 MCF7	IC_50_ (µM) 42.67 44.90	[20]
*E. dendroides*	23*R*/*S*-3β-hydroxycycloart-24-ene-23-methyl ether (141)	MTT assay HepG2 Huh-7 KLM-1 1321N1 HeLa	IC_50_ (µM) 20.67 16.24 22.59 25.99 40.50	[21]
	24-methylene cycloartan-3β-ol (142)	HepG2 Huh-7 KLM-1 1321N1 HeLa	10.93 7.42 21.48 12.32 13.68	
	Cycloart-23-*ene*-3β,25-diol monoacetate (143)	HepG2 Huh-7 KLM-1 1321N1	12.81 <0.47 22.48 25.17	
	3β-hydroxy-cycloart-23-*ene*-25 methyl ether (144)	HepG2 Huh-7 KLM-1 1321N1 HeLa	12.72 <0.44 <0.44 0.63 3.7	
	24*R*/*S*-3β-hydroxy-25-methylenecycloartan-24-ol (145)	HepG2 Huh-7 KLM-1 1321N1 HeLa	15.54 16.33 22.38 13.53 >4.52	
*E. denticulata*	12-taraxast-3β, 19, 21 (α)-triol (146)	MTT assay DU-145	IC_50_ (µM) 12.2 27.5 18.3	[22]
	Cycloartane-3, 25-diol (147)			
	Cycloartane-3,24, 25-triol (148)			
*E. ebracteolata*	Euphebracteolatin C (149)	CCK-8 assay HepG2 MCF7 A549	IC_50_ µM 14.29 34.81 40.85	[23]
	Euphebracteolatin D (150)	HepG2 MCF7 A549	23.69 28.62 39.25	
	Euphebracteolatin E (151)	HepG2 MCF7 A549	38.96 29.67 36.27	
	Euphorpekone B (152)	HepG2 MCF7 A549	12.33 25.29 38.82	
	Jolkinolide B (136)	MTS assay HL-60 SMMC-7721 A549 MCF-7 SW480	IC_50_ (µM) 5.2 3.8 11.9 16.2 10.2	[25]
	Euphoroid B (153)	MTT assay A549	IC_50_ (µM) 22.87	[26]
	Euphoroid C (154)	A549 MCF-7 Lovo HepG2	28.7 28.57 27.0 28.0	
	Jolkinolide A (155)	A549	18.56	
*E. fischeriana*	12-deoxyphorbol-13-(9*Z*,12*Z*)-octadecadienoate (156)	MTT assay HeLa HepG2	IC_50_ (µM) 3.54 8.32	[28]
	12-deoxyphorbol-13-dimethylpentadecanoate (157)	HeLa HepG2	5.72 11.45	
	Euphonoid H (158)	MTT Assay MDA-MB-231 HCT-15 RKO C4-2B C4-2B/ENZR	IC_50_ (µM) 21.8 28.57 20.46 5.52 4.16	[29]
	Euphonoid I (159)	MDA-MB-231 HCT-15 RKO C4-2B C4-2B/ENZR	7.95 12.45 8.78 4.49 5.74	
	Raserrane A (160)	C4-2B	34.09	
	Raserrane B (161)	C4-2B C4-2B/ENZR	23.34 36.98	
	Fischerianin A (162)	MTT assay HepG2 A375 HL-60 K562 HeLa	IC_50_ (µM) 17.59 21.46 15.59 14.99 13.24	[31]
	Fischerianin B (163)	HepG2 A375 HL-60 K562 HeLa	11.23 18.34 12.82 17.82 5.31	
	Langduin A (164)	HepG2 A375 HL-60 K562 HeLa	14.47 13.34 20.18 13.28 19.36	
	Langduin A6 (165)	HepG2 A375 HL-60 K562 HeLa	16.55 9.64 21.03 8.46 11.57	
	Euphonoid A (166)	MTT assay C4-2B C4-2B/ENZR HCT-15 RKO	IC_50_ (µM) 9.18 9.70 18.3 16.2	[32]
	Euphonoid B (167)	C4-2B C4-2B/ENZR RKO	13.4 11.1 35.1	
	Euphonoid C (168)	C4-2B C4-2B/ENZR HCT-15 RKO	17.7 15.2 13.4 21.3	
	Euphonoid D (169)	C4-2B C4-2B/ENZR HCT-15 RKO	9.23 15.1 23.2 34.4	
	Euphonoid E (170)	C4-2B C4-2B/ENZR	16.1 22.1	
	Euphonoid F (171)	C4-2B C4-2B/ENZR	24.9 40.1	
	Euphonoid G (172)	C4-2B C4-2B/ENZR	18.1 20.1	
	Euphonoid H (158)	C4-2B C4-2B/ENZR HCT-15 RKO	7.39 9.20 19.0 22.9	
	Raserrane B (161)	C4-2B C4-2B/ENZR HCT-15 RKO	16.3 16.4 28.2 42.1	
	11-oxo-ebracteolatanolide B (173)	C4-2B C4-2B/ENZR HCT-15 MDA-MB-231	2.85 2.42 15.2 14.5	
	Caudicifolin (174)	C4-2B C4-2B/ENZR HCT-15 RKO MDA-MB-231	2.22 5.39 12.6 15.3 8.81	
	Jolkinolide A (155)	C4-2B C4-2B/ENZR	10.1 16.1	
	17-hydroxyjolkinolide B (175)	C4-2B C4-2B/ENZR	12.3 14.0	
	Jolkinolide B (136)	C4-2B C4-2B/ENZR HCT-15 RKO MDA-MB-231	4.43 5.89 47.9 35.8 30.7	
	Methyl-8,11-3-dihydroxy-12- oxo-*ent*-abietadi-13,15(17)-ene-16-oate (176)	C4-2B C4-2B/ENZR HCT-15 RKO MDA-MB-231	4.95 4.27 25.6 23.3 23.8	
	7-dehydroabietanone (177)	C4-2B C4-2B/ENZR	14.2 29.9	
	Abieta-8,11,13-triene (178)	C4-2B C4-2B/ENZR	20.1 37.1	
	15-hydroxydehydroabietic acid (179)	C4-2B	33.1	
	(4α*S*,10α*S*)-1,2,3,4,4α,10α-hexahydro-1,1,4α-trimethyl-7-(1-methyl)phenanthrene (180)	C4-2B C4-2B/ENZR	36.2 26.2	
	2-phenanthrenyl] ethanone (181)	C4-2B	34.0	
	(4β*S*,8α*S*)-2-phenanthrenecarboxylic acid,4β,5,6,7,8,8α,9,10-octahydro-3-hydroxy-4β,8,8-trimethyl-methyl ester (182)	C4-2B	23.1	
	Isopimara-7,15-dien-3-one (183)	C4-2B C4-2B/ENZR	21.9 24.2	
	Araucarol (184)	C4-2B C4-2B/ENZR	19.2 34.3	
	Araucarone (185)	C4-2B C4-2B/ENZR HCT-15	16.0 24.1 47.1	
	Ent-3β, (13*S*)-dihydroxyatis-16- en-14-one (186)	C4-2B C4-2B/ENZR	13.2 25.3	
	Ent-(13*R*,14*R*)-13,14-dihydroxyatis-16-en-3-one (187)	C4-2B C4-2B/ENZR HCT-15	18.8 15.2 39.2	
	Ent-atis-16-ene-3,14-dione (188)	C4-2B	26.7	
	Ent-(13*S*)-13-hydroxyatis- 16-ene-3,14-dione (189)	C4-2B	30.5	
	3-oxoatisane-16α,17-diol (190)	C4-2B C4-2B/ENZR	23.7 29.1	
	3α-hydroxy-ent-16-kauren (191)	C4-2B	26.2	
	Ent-kaurane-3β,16β,17-triol (192)	C4-2B/ENZR HCT-15	21.7 28.1	
	Ent-16β-*H*-3-oxokauran-17-ol (193)	C4-2B C4-2B/ENZR	22.8 20.1	
	Ent-kaurane-3-oxo-16β,17-diol (194)	C4-2B C4-2B/ENZR HCT-15	17.0 23.0 43.2	
	Fischerianoid A (195)	MTT assay MM-231 SMMC-7721 HEP3B	IC_50_ (µM) 12.10 32.48 15.95	[33]
	Fischerianoid B (196)	HL-60 MM-231 HEP3B SW-480	28.78 9.12 8.50 35.52	
	Fischerianoid C (197)	MM-231 HEP3B	25.45 27.34	
*E. gedrosiaca*	13β-*O*-propanoyl-5α-*O*-methylbutanoyl-7α,13β-*O*-diacetyl-17α-*O*-nicotinoyl-14-oxopremyrsinane (198)	MTT assay MDA-MB-231 MCF-7	IC_50_ (µM) 10.8 22.2	[35]
	3β-*O*-propanoyl-5α-*O*-benzoyl-7α,13β, 17α-*O*-triacetyl-14-oxopremyrsinane (199)	MDA-MB-231 MCF-7	22.2 27.8	
	3β-*O*-propanoyl-5α-*O*-isobutanoyl-7α,13β,17α-*O*-triacetyl-14-oxopremyrsinane (200)	MDA-MB-231	24.5	
	3β-*O*-propanoyl-5α-*O*-isobutanoyl-7α,13β-*O*-diacetyl-17α-*O*-nicotinoyl-14-oxopremyrsinane (201)	MDA-MB-231	27.3	
	2,5,7,10,15-*O*-pentaacetyl-3-*O*-propanoyl-14-*O*-benzoyl-13,17-epoxy-8-myrsinene (202)	MDA-MB-231	33.7	
*E. glomerulans*	Euphoglomeruphane H (203)	MTT assay MCF-7/ADR	IC_50_ (µM) 39.3	[36]
*E. grandicornis*	Hexyl(*E*)-3-(4-hydroxy-3-methoxyphenyl)-2-propenoate (204)	MTT assay MCF-7 HCC70	IC_50_ (µM) 23.41 29.45	[37]
	6-Angeloyloxy-20-acetoxy-13-isobutanoyloxy-4,9-dihydroxytiglia-1,6-dien-3-one (205)	MTT assay A549	Cell viability (%) 49.2	[38]
*E. grantii*	Eupha-8,24-dien-3β-ol (Euphol) (206)	SRB assay MCF-7 MCF-7ADR	IC_50_ (µM) 26.25 27.77	[39]
	Cycloartenyl acetate (207)	MCF-7 MCF-7ADR	25.3 18.56	
	Cycloartenol (208)	MCF-7 MCF-7ADR	23.73 15.6	
	Epifriedelinyl acetate (209)	MCF-7 MCF-7ADR	26.18 19.04	
	Euphylbenzoate (210)	MCF-7 MCF-7ADR	3.47 3.22	
		Flow cytometry	The death is induced by apoptosis	
*E. helioscopia*	Euphohelinoid A (211)	SRB assay HepG2 HeLa HL-60 SMMC-7221	IC_50_ (µM) 24.3 28.4 18.6 29.6	[41]
	Euphohelinoid B (212)	HepG2 HeLa HL-60 SMMC-7221	10.2 9.3 8.1 9.8	
	Euphohelinoid D (213)	HeLa HL-60 SMMC-7221	34.5 34.1 30.1	
	Euphohelinoid F (214)	HepG2 HeLa HL-60 SMMC-7221	12.5 14.1 13.3 11.1	
	Euphornin L (215)	HepG2 HeLa HL-60 SMMC-7221	22.8 25.7 13.1 14.3	
	Helioscopianoid O (216)	HeLa HL-60 SMMC-7221	26.2 18.2 19.5	
	Euphoscopin I (217)	HepG2 HeLa HL-60 SMMC-7221	24.1 29.7 14.3 18.7	
	Euphoscopin J (218)	HepG2 HeLa HL-60 SMMC-7221	14.9 13.7 12.4 15.0	
	Euphoscopin B (219)	HepG2 HeLa HL-60 SMMC-7221	23.3 29.2 20.2 27.1	
	Euphelionolide F (220)	MTT assay MCF-7 PANC-1	IC_50_ (µM) 9.5 10.7	[42]
	Euphelionolide L (221)	MCF-7 PANC-1	9.8 10.3	
*E. hypericifolia*	Euphypenoid A (222)	MTT assay HCT-116	IC_50_ (µM) 12.8	[43]
	20(*S*),24(*R*)-20,24-epoxy-24-methyldammaran-3β-ol (223)	HCT-116	26.8	
	(23*E*)-25-methoxycycloart-23-en- 3-one (224)	HCT-116	7.4	
	Isomotiol (225)	HCT-116	10.6	
*E. kansuensis*	Euphorboside A (226)	MTT assay RKO MDAMB-231 A375 8 HCT-15 HCT-15/5-FU A549 A549/CDDP HepG2 HepG2/DOX	IC_50_ (µM) 3.70 4.15 8.27 14.7 15.0 16.2 16.4 18.8 33.2	[45]
*E. kansui*	Wilfoside KIN (227)	MTT Assay HepG2 MCF7	IC_50_ (µM) 12.55 >20	[47]
	Cynsaccatol L (50)	HepG2 MCF7	12.61 >20	
	Kanesulone A (228)	HepG2 MCF7	18.24 >20	
	3β,7β,15β-triacetyloxy-5α-benzoyloxy-2α,8α-dihydroxyjatropha-6(17),11*E*-diene-9, 14-dione (229)	HepG2 MCF7	18.26 >20	
	13-hydroxyingenol-3-(2,3-dimethylbutanoate)-13-dodecanoate (230)	HepG2 MCF7 GSC3 GSC12 293T HAC T98G	>20 17.12 1.67 2.75 21.93 19.23 16.77	
	Euphol (206)	GSC-3 GSC-12	8.89 13.0	
	Lucidal (231)	GSC-3 GSC-12 293T HAC T98G	4.71 3.25 21.07 30.22 20.77	
*E. kopetdaghi*	14-Nicotinyl-3,5,10,15,17-pentaacetyl-8-isobutanoyl-cyclomyrsinol-7- one (Kopetdaghinane A) (232)	MTT assay MCF-7	IC_50_ (µM) 38.10	[49]
		OVCAR-3	51.23	
*E. lactea*	Friedelan-3β-ol (233)	HN22 Flow cytometry	It induced an S-phase cell cycle arrest	[50]
*E. lathyris*	Euphorbia Factor L1 (68)	Tumour induced by Mouse 4T1 in BALB/c	Decreased the generation of IL-β, IL-6, TNF-α	[51]
		ELISA	Downregulated DDR1 protein expression and immuno-reactivity in SHI mice	
		Western blot Flow cytometry	No differences were detected in CD4+, CD8+, CD49b+ T cells, and Tregs between the DDR1-OE group and the DDR1-OE+EFL1 group	
	15β-hydroxy-5α-acetoxy-3α-benzoyloxy-7β-nicotinoyloxylathyol (234)	MTT assay MCF-7 HepG2	IC_50_ (µM) 9.43 13.22	[57]
	Euphorbia Factor L2 (80)	MTT assay KB KB-VIN	IC_50_ (µM) 33.2 7.2	[58]
	Euphorbia Factor L3 (69)	A549 MDA-MB-231 KB KB-VIN MCF-7	14.6 31.6 7.9 8.0 25.9	
	Euphorbia Factor L8 (83)	A549 MDA-MB-231 KB KB-VIN MCF-7	11.8 24.4 17.7 16.9 23.8	
	Euphorbia Factor L9 (74)	A549 MDA-MB-231 KB KB-VIN MCF-7	6.7 21.9 6.1 5.7 8.4	
	Euphorbia Factor L24 (87)	MTT assay HCT116 MCF-7 786-0 HepG2	IC_50_ (µM) 6.44 8.43 15.3 9.32	[59]
*E. microsphaera*	(3a*R*,4*S*,4a*S*,5*R*,7a*S*,9a*S*)-5-hydroxy-5,8-dimethyl-3-methylene-2-oxo- 2,3,3a,4,4a,5,6,7,7a,9a-decahydroazuleno [6,5-b] furan-4-yl acetate (Aryanin) (235)	MTT assay MCF7 24 h 72 h	IC_50_ (µg/mL) 13.81 49.35	[63]
*E. neriifolia*	Neritriterpenols A (236)	MTT assay Hep G2	IC_50_ (µM) 25.9	[65]
	(+)-(24*R*)-3β,24,25-trihydroxyeuph-8-en-7-one (Neritriterpenol B) (237)	WiDR HepG2	47.2 44.0	
	Neritriterpenol E (238)	A549 WiDR HepG2	45.7 32.3 35.9	
	(+)-(23*R*,24*R*)-epoxy-3α,25-dihydroxyeuph-8-en-7-one (Neritriterpenol F) (239)	HepG2	39.4	
	(+)-(24*R*)-24,25-dihydroxyeuph-8-en-3,7-dione (Neritriterpenol G) (240)	WiDR HepG2	48.9 36.6	
	(23*E*)-eupha-8,23-diene-3β,25-diol-7-one (117)	A549 WiDR HepG2	25.5 20.5 37.6	
	(+)-(24*S*)-eupha-8,25-diene-3β,24-diol-7-one (118)	A549 WiDR MCF7 HepG2	23.8 20.8 32.3 15.2	
	(24*R*)-eupha-8,25-diene-3β,24-diol-7-one (119)	A549 WiDR MCF7 HepG2	20.4 17.1 30.7 12.2	
	Sooneuphanone B (116)	A549 WiDR MCF7 HepG2	12.8 23.3 17.9 8.0	
	Phonerilin B (241)	SRB assay A549 HL-60	IC_50_ (µM) 8.6 9.1	[66]
	Phonerilin E (242)	A549 HL-60	4.9 9.2	
	Phonerilin F (243)	A549 HL-60	3.8 4.5	
	Phonerilin H (244)	A549 HL-60	7.5 5.7	
	20-*O*-diacetyl-ingenol (245)	HL-60	3.1	
	7,12-*O*-diacetyl-8-*O*-tigloylingol (246)	A549 HL-60	6.4 9.5	
	Ent-atisane-3α,16α,17-triol (247)	MTT assay HepG2 HepG2/Adr	IC_50_ (µM) 13.7 15.57	[67]
	(4*R*,5*S*,8*S*,9*R*,10*S*,13*R*,16*S*)- ent-16α,17-dihydroxy-19-tigloyloxykauran-3-one (248)	HepG2	0.01	
*E. pedroi*	Spiropedroxodiol (95)	MTT assay L5178Y-PAR L5178Y-MDR Colo205 Colo320	IC_50_ (µM) 42.3 46.8 16.8 27.7	[68]
	β-sitostenone (249)	Colo 205 Colo320	46.6 21.3	
	Cycloart-23-ene-3β,25-diol (250)	L5178Y-PAR Colo 205 Colo320 MRC-5	49.4 16.7 31.6 12.9	
	Helioscopinolide E (251)	L5178Y-PAR	32.9	
*E. pekinensis*	(11*R*,12*S*)-2,11,12-trihydroxy-ent-isopimara-1,7,15-trien-3-one (252)	CCK8 method U-937 LOVO	IC_50_ (µM) 25.1 27.7	[69]
	Isopimara-7,15-dien-3β-ol (253)	K-562	0.87	
	Eupneria R (254)	U-937 LOVO	30.5 27	
	Euphodane A (255)	U-937 LOVO K-562	5.9 26.8 32.2	
	Euphodane B (256)	U-937 LOVO	36.7 35.03	
	Euphodane C (257)	U-937 LOVO K-562	24.5 39.3 31.3	
	Euphodane D (258)	U-937 LOVO	25.1 29.7	
	Jolkinol B (259)	U-937 LOVO K-562	3.6 8.44 25.3	
*E. saudiarabica*	Glutinol (260)	MTT assay MCF-7 Flow cytometry	IC_50_ (µM) 9.83 Induced apoptosis	[73]
*E. schimperiana*	3,30-di-*O*-methylellagic acid (261)	MTT assay PC3	IC_50_ (µg/mL) 5.5	[74]
*E. sororia*	Euphosorophane I (262)	P-gp ATPase activity assay	This compound reversed P-gp-mediated MDR cell (multidrug resistance) by inhibiting the ABCB1 drug efflux function in drug-resistant MCF-7/ADR cells	[75]
*E. stracheyi*	3-*O*-benzoyl-20-deoxymgenol (263)	MTT assay HL-60 A-549 SMMC-7721 MCF-7 SW480	IC_50_ (µM) 10.5 21.47 18.36 18.82 16.25	[76]
*E. tirucalli*	Tirucadalenone (264)	MTT assay K562	IC_50_ (μg/mL) 22	[78]
	Euphol (206)	MTT assay U87-MG U373 U251 GAMG SW1088 SW1783 SNB19 RES186 RES259 KNS42 UW479 SF188 HCB2 HCB149	IC_50_ (µM) 26.41 30.48 29.01 8.73 27.12 19.62 31.05 16.70 10.34 19.94 15.26 5.98 11.66 21.68	[79]
	Euphol (206)	MTS assay T47D MDA-MB-231 MDA-MB-468 BT20 HS587T MCF-7 MCF7/AZ JHU-O22 HN13 SCC25 SCC4 SCC14 FADU SW480 SW620 CO115 HCT15 HT29 SK-CO-10 DLD1 LOVO DIFI Caco2 U87-MG U373 U251 GAMG SW1088 SW1783 RES186 RES259 KNS42 UW479 SF188 PC-3 LNCaP T24 5637 HT1376 MCR DAOY ONS76 JEG3 A431 H292 SKMES1 A549 SK-LU-1 SIHA CASKI C33A HELA KYSE30 KYSE70 KYSE270 KYSE410 Mia PaCa-2 PANC-1 PSN-1 BXPC-3 Capan-1 COLO858 COLO679 A375 WM1617 WM9 WM852 WM278 WM35 WN793 SKMEL-37 PA-1 SW626	IC_50_ (µM) 38.89 9.08 30.89 8.96 18.15 18.76 33.42 26.35 8.89 6.65 19.82 15.81 20.17 5.79 10.02 9.58 5.47 6.52 17.53 2.56 11.49 11.38 35.19 26.41 30.48 29.01 8.73 27.12 19.62 16.70 10.34 19.94 15.26 5.98 11.95 1.41 30.72 4.83 25.25 7.40 5.72 21.72 16.65 17.79 13.25 25.62 11.01 22.83 24.74 24.74 21.32 17.55 3.52 8.77 10.71 4.35 8.46 21.47 3.71 5.47 16.33 14.02 8.93 9.67 16.32 9.67 7.61 27.46 12.40 5.96 10.07 7.97 30.40	[80]
*E. umbellata*	Euphol (206)	MTT assay K-562 HL-70	IC_50_ (µM) 34.44 39.98	[81]

**MTT:** 3-[4,5-dimethylthiazol-2-yl]-2,5 diphenyl tetrazolium bromide; **MTS:** 3-(4, 5-dimethylthiazol-2-yl)-5-(3-carboxymethoxyphenyl)- 2-(4-sulfophenyl)- H-tetrazolium; **SRB:** sulforhodamine B assay; **5637:** carcinoma from the urinary bladder; **1321N1:** astrocytoma (malignant gliomas); **293T:** clone derivative of the human embryonic kidney (HEK) 293 cell line; **A375:** melanoma; **A431:** squamous carcinoma; **A549:** lung cancer; **A549/CDDP:** Cisplatin resistance in lung cancer; **BT20:** breast cancer; **BXPC-3:** pancreatic adenocarcinoma; **C33A:** cervical cancer; **C4-2B:** prostate cancer; **C4-2B/ENZR:** prostate cancer enzalutamide resistant; **Caco2:** colon cancer; **Capan-1:** pancreatic adenocarcinoma; **CASKI:** epithelial cell from the cervix with epidermoid; **CO115:** colon carcinoma in vitro from solid xenografts; **Colo205:** colon carcinoma; **Colo320:** colon carcinoma; **COLO679:** skin melanoma; **COLO858:** skin melanoma; **DAOY:** medulloblastoma; **DIFI:** colorectal cancer; **DLD1:** colorectal adenocarcinoma; **DU-145:** prostate cancer; **FADU:** hypopharyngeal carcinoma; **GAMG:** glioblastoma; **GSC12:** glioma; **GSC3:** glioma; **H292:** pulmonary mucoepidermoid carcinoma; **HAC:** ovarian adenocarcinoma; **HCB149:** immortalized glioma; **HCB2**: Primary Glioma; **HCC70:** epithelial cell from primary ductal carcinoma; **HCT116:** colon cancer; **HCT-15:** colorectal adenocarcinoma; **HCT-15/5-FU 5-:** Fluorouracil Resistance in Colon Cancer; **HeLa:** Cervix Adenocarcinoma; **HEP3B:** hepatoma; **HepG2:** Hepatocarcinoma; **HepG2/Adr:** hepatoblastoma adriamycin resistant; **HepG2/DOX:** hepatoblastoma doxorubicin resistant; **HL-60:** promyelocytic leukemia; **HL-70**: lymphoblast promyeolocytic leukemia; **HN13:** squamous cell carcinoma of the oral tongue; **HS587T:** carcinoma of the breast; **HT1376:** urinary bladder carcinoma; **HT29:** colorectal adenocarcinoma; **Huh-7:** hepatoma; **JEG3:** choriocarcinoma; **JHU-O22:** Laryngeal carcinoma; **K562:** chronic myelogenous leukemia; **KB:** epithelial carcinoma; **KB-VIN:** epithelial carcinoma vincristine resistant; **KLM-1:** pancreatic cancer; **KNS42:** glioma; **KYSE270:** esophageal squamous carcinoma; **KYSE30:** squamous carcinoma; **KYSE410:** esophageal carcinoma; **KYSE70:** esophageal carcinoma; **L5178Y-MDR:** lymphoma multidrug resistant; **L5178Y-PAR:** lymphoma parental; **LNCaP:** prostate carcinoma; **Lovo:** prostate carcinoma; **MCF-7:** breast cancer; **MCF-7ADR:** breast cancer adriamycin resistant; **MCF7/AZ:** breast cancer; **MCR:** bladder cancer; **MDA-MB-231:** human breast cancer cell line; **MDA-MB-468:** breast cancer; **Mia PaCa-2:** pancreas carcinoma; **MM-231:** breast cancer; **MRC-5:** lung fibroblast (breast cancer); **ONS76:** medulloblastoma; **OVCAR-3:** ovarian adenocarcinoma; **PA-1:** ovarian teratocarcinoma; **PANC-1:** pancreatic carcinoma; **PC-3:** prostatic adenocarcinoma; **PSN-1:** pancreatic carcinoma; **RES186:** glioma; **RES259:** glioma; **RKO:** colon carcinoma; **SCC14:** head and neck squamous cell carcinoma cell lines; **SCC-25:** tongue squamous cell carcinoma; **SCC4:** tongue squamous cell carcinoma; **SF188:** glioblastoma; **SIHA:** uterine squamous cell carcinoma; **SK-CO-10:** colon cancer; **SK-LU-1:** lung adenocarcinoma; **SKMEL-37:** melanoma; **SKMES1:** lungs squamous cell carcinoma; **SMMC-7721:** hepatocellular carcinoma; **SNB19:** glioblastoma; **SW1088:** brain astrocytoma; **SW1783:** brain astrocytoma; **SW480:** colon cancer; **SW620:** colorectal cancer; **SW626:** ovary adenocarcinoma; **T24:** urinary bladder carcinoma; **T47D:** breast cancer; **T98G:** glioblastoma; **U251:** glioblastoma; **U373:** glioblastoma astrocytoma; **U87-MG:** glioblastoma; **U-937:** histiocytic lymphoma; **UW479:** glioma; **WiDR:** colorectal adenocarcinoma; **WM1617:** melanoma; **WM278:** melanoma; **WM35:** melanoma; **WM852:** melanoma; **WM9:** melanoma; **WN793:** melanoma.

## Data Availability

Not applicable.

## References

[1] González-Costa M., Padrón-González A.A. (2019). La inflamación desde una perspectiva inmunológica: Desafío a la Medicina en el siglo XXl. Rev. Haban Cienc. Méd..

[2] Kumar V., Abbas A.K., Fausto N., Mitchell R.N. (2018). Robbins Basic Pathology.

[3] Yang H.Z., Wang J.P., Mi S., Liu H.Z., Cui B., Yan H.M., Lu W. (2012). TLR4 activity is required in the resolution of pulmonary inflammation and fibrosis after acute and chronic lung injury. Am. J. Pathol..

[4] Oscanoa-Espinoza T., Lizaraso-Soto F. (2015). Antiinflamatorios no esteroides: Seguridad gastrointestinal, cardiovascular y renal. Rev. Gastroenterol. Perú.

[5] Gordon S. (2003). Alternative activation of macrophages. Nat. Rev. Immunol..

[6] Fernandes J.V., Cobucci R.N., Jatobá C.A., Fernandes T.A., de Azevedo J.W., de Araújo J.M. (2015). The Role of the Mediators of Inflammation in Cancer Development. Pathol. Oncol. Res..

[7] Campos-Xolalpa N., Alonso-Castro Á.J., Sánchez-Mendoza E., Zavala-Sánchez M.A., Pérez-Gutiérrez S. (2017). Cytotoxic activity of the chloroform extract and four diterpenes isolated from Salvia ballotiflora. Rev. Bras. Farmacogn..

[8] Virshette S.J., Patil M.K., Somkuwar A.P. (2019). A review on medicinal plants used as anti-inflammatory agents. J. Pharmacogn. Phytochem..

[9] Schlaepfer L., Mendoza E.J.A. (2010). Las Plantas Medicinales en la Lucha Contra el cáNcer, Relevancia Para México. Rev. Mex. Cienc. Farm..

[10] Bittner M., Alarcón J., Aqueveque P., Becerra J., Hernández V., Hoeneise M., Silva M. (2001). Estudio químico de especies de la familia Euphorbiaceae en Chile. Bol. Soc. Chil. Quim..

[11] Amtaghri S., Akdad M., Slaoui M., Eddouks M. (2022). Traditional uses, pharmacological, and phytochemical studies of *Euphorbia*: A review. Curr. Top. Med. Chem..

[12] Li Y.N., He J., Zhang J., Shi Y.X., Guo L.B., Peng Z.C., Xu J.K. (2021). Existing knowledge on *Euphorbia fischeriana* Steud (Euphorbiaceae): Traditional uses, clinical applications, phytochemistry, pharmacology and toxicology. J. Ethnopharmacol..

[13] Martínez G.M., Jiménez R.J., Cruz D.R., Juárez A.E., García R., Cervantes A., Mejía H.R. (2002). Los géneros de la familia Euphorbiaceae en México. (Parte A). An. Inst. Biol..

[14] Xu Y., Tang P., Zhu M., Wang Y., Sun D., Li H., Chen L. (2021). Diterpenoids from the genus *Euphorbia*: Structure and biological activity (2013–2019). Phytochemistry.

[15] Zhao H., Sun L., Kong C., Mei W., Dai H., Xu F., Huang S. (2022). Phytochemical and pharmacological review of diterpenoids from the genus *Euphorbia* Linn (2012–2021). J. Ethnopharmacol..

[16] Rozimamat R., Hu R., Aisa H.A. (2018). New isopimarane diterpenes and nortriterpene with cytotoxic activity from *Euphorbia alatavica* Boiss. Fitoterapia.

[17] Choodej S., Hanthanong S., Aree T., Pudhom K. (2020). Diterpenoids from the aerial parts of *Euphorbia antiquorum* and their efficacy on nitric oxide inhibition. Phytochemistry.

[18] An L., Liang Y., Yang X., Wang H., Zhang J., Tuerhong M., Li D., Wang C., Lee D., Xu J. (2019). NO inhibitory diterpenoids as potential anti-inflammatory agents from *Euphorbia antiquorum*. Bioorg. Chem..

[19] Zhao H., Duan R.J., Kong C.H., Dai H.F., Mei W.L., Xu F.Q., Huang S.Z. (2023). Two new anti-inflammatory trachylobane diterpenoids from *Euphorbia atoto*. J. Asian Nat. Prod. Res..

[20] Aljubiri S.M., Mahgoub S.A., Almansour A.I., Shaaban M., Shaker K.H. (2021). Isolation of diverse bioactive compounds from *Euphorbia balsamifera*: Cytotoxicity and antibacterial activity studies. Saudi J. Biol. Sci..

[21] Hassan A.R., Ashour A., Amen Y., Nagata M., El-Toumy S.A., Shimizu K. (2022). A new cycloartane triterpene and other phytoconstituents from the aerial parts of *Euphorbia dendroides*. Nat. Prod. Res..

[22] Shamsabadipour S., Zarei S.M., Ghanadian M., Ayatollahi S.A., Rahimnejad M.R., Saeedi H., Aghaei M. (2018). A New Taraxastane triterpene from *Euphorbia Denticulata* with cytotoxic activity against prostate cancer cells. Iran. J. Pharm. Res..

[23] Ding K., Zhang Y.Y., Yang T., Lian W.W., Xia C., Wang W.P., Zhang W.K., He J., Xu J.K. (2023). New rosane diterpenoids and their analogs from *Euphorbia ebracteolata* Hayata. Chem. Biodivers..

[24] Chun J., Mah S.Y., Kim Y.S. (2023). Anti-inflammatory effect of ebractenoid f, a major active compound of *Euphorbia ebracteolata* Hayata, through inhibition of nuclear Factor-κB activation. Plants.

[25] Ma Y.L., Tang X.H., Yuan W.J., Ding X., Di Y.T., Hao X.J. (2018). Abietane diterpernoids from the roots of *Euphorbia ebracteolata*. Nat. Prod. Bioprospect..

[26] Han C., Peng Y., Wang Y., Huo X., Zhang B., Li D., Leng A., Zhang H., Ma X., Wang C. (2018). Cytotoxic ent-Abietane-type diterpenoids from the roots of *Euphorbia ebracteolata*. Bioorg. Chem..

[27] Bai J., Huang X.Y., Liu Z.G., Gong C., Li X.Y., Li D.H., Hua H.M., Li Z.L. (2018). Four new compounds from the roots of *Euphorbia ebracteolata* and their inhibitory effect on LPS-induced NO production. Fitoterapia.

[28] Ma X.M., Mo L.Y., Ren Z.P., Fan X.N., Sun P.H., Tian H.Y., Yang N., Zi J.C. (2023). New abietane and tigliane diterpenoids from the roots of *Euphorbia fischeriana* and their cytotoxic activities. J. Asian Nat. Prod. Res..

[29] Zhu Q.F., Xu G.B., Liao S.G., Yan X.L. (2022). Ent-Abietane diterpenoids from *Euphorbia fischeriana* and their cytotoxic activities. Molecules.

[30] Sun C.P., Chang Y.B., Wang C., Lv X., Zhou W.Y., Tian X.G., Zhao W.Y., Ma X.C. (2021). Bisfischoids A and B, dimeric ent-abietane-type diterpenoids with anti-inflammatory potential from *Euphorbia fischeriana* Steud. Bioorg. Chem..

[31] Xie R., Xia G., Zhu J., Lin P., Fan X., Zi J. (2021). Daphnane-type diterpenoids from *Euphorbia fischeriana* Steud and their cytotoxic activities. Fitoterapia.

[32] Yan X.L., Zhang J.S., Huang J.L., Zhang Y., Chen J.Q., Tang G.H., Yin S. (2019). Euphonoids A-G, cytotoxic diterpenoids from *Euphorbia fischeriana*. Phytochemistry.

[33] Li M., He F., Zhou Y., Wang M., Tao P., Tu Q., Lv G., Chen X. (2019). Three new ent-abietane diterpenoids from the roots of *Euphorbia fischeriana* and their cytotoxicity in human tumor cell lines. Arch. Pharm. Res..

[34] Lan Y.H., Chen I.H., Lu H.H., Guo T.J., Hwang T.L., Leu Y.L. (2022). Euphormins A and B, new pyranocoumarin derivatives from *Euphorbia formosana* Hayata, and their anti-inflammatory activity. Molecules.

[35] Yazdiniapour Z., Sohrabi M.H., Motinia N., Zolfaghari B., Mehdifar P., Ghanadian M., Lanzotti V. (2023). Diterpenoids from *Euphorbia gedrosiaca* as potential anti-proliferative agents against breast cancer cells. Metabolites.

[36] Hasan A., Liu G.Y., Hu R., Aisa H.A. (2019). Jatrophane Diterpenoids from *Euphorbia glomerulans*. J. Nat. Prod..

[37] Kemboi D., Peter X., Langat M.K., Mhlanga R., Vukea N., de la Mare J.A., Noundou S.X., Krause R.W.M., Tembu V.J. (2021). In vitro cytotoxic effects of chemical constituents of *Euphorbia grandicornis* Blanc against breast cancer cells. Sci. Afr..

[38] Tsai J.Y., Rédei D., Hohmann J., Wu C.C. (2020). 12-Deoxyphorbol esters induce growth arrest and apoptosis in human lung cancer A549 cells via activation of PKC-δ/PKD/ERK signalling pathway. Int. J. Mol. Sci..

[39] Radi M.H., El-Shiekh R.A., El-Halawany A.M., Al-Abd A.M., Abdel-Sattar E. (2023). In vitro cytotoxic study of *Euphorbia grantii* Oliv. aerial parts against MCF-7 and MCF-7ADR breast cancer cell lines: A bioactivity-guided isolation. ACS Omega.

[40] Yang H.Y., Yao W., Huang P.Z., Xu H., Ma Q., Chen X., Chen J.J., Gao K. (2023). Euphohelides A-C, ent-abietane-type norditerpene lactones from *Euphorbia helioscopia* and their anti-inflammatory activities. J. Nat. Prod..

[41] Lu Y.B., Luo S., Wang Y.X., Feng Z.Y., Gao K., Chen J.J. (2022). Jatrophane diterpenoids with cytotoxic activity from the whole plant of *Euphorbia helioscopia* L.. Phytochemistry.

[42] Wang W.P., Jiang K., Zhang P., Shen K.K., Qu S.J., Yu X.P., Tan C.H. (2018). Highly oxygenated and structurally diverse diterpenoids from *Euphorbia helioscopia*. Phytochemistry.

[43] Hu R., Sang J., Li W., Tian Y., Zou M.F., Tang G.H., Yin S. (2021). Structurally diverse triterpenoids with cytotoxicity from *Euphorbia hypericifolia*. Fitoterapia.

[44] Xue L.Y., Chen B.L., Yuan F.Y., Zhu Q.F., Zhang X., Lin Y., Long Q.D., Liao S.G. (2022). Six new tigliane diterpenoids with anti-inflammatory activity from *Euphorbia kansuensis*. Arab. J. Chem..

[45] Yan X.L., Sang J., Zhang X., Lin Y., Long Q.D., Zhu Q.F., Liao S.G. (2021). Euphorboside A, a cytotoxic meroterpenoid glycoside with an unusual humulene-phloroglucinol skeleton from *Euphorbia kansuensis*. Fitoterapia.

[46] Feng X., Li J., Li H., Chen X., Liu D., Li R. (2023). Bioactive C21 steroidal glycosides from *Euphorbia kansui* promoted HepG2 cell apoptosis via the degradation of ATP1A1 and inhibited macrophage polarization under co-cultivation. Molecules.

[47] Li J.C., Li S.Y., Tang J.X., Liu D., Feng X.Y., Rao K.R., Zhao X.D., Li H.M., Li R.T. (2022). Triterpenoids, steroids and other constituents from *Euphorbia kansui* and their anti-inflammatory and anti-tumour properties. Phytochemistry.

[48] Zhang J.S., Weng H.Z., Huang J.L., Tang G.H., Yin S. (2018). Anti-inflammatory ingenane diterpenoids from the roots of *Euphorbia kansui*. Planta Med..

[49] Riahi F., Dashti N., Ghanadian M., Aghaei M., Faez F., Jafari S.M., Zargar N. (2020). Kopetdaghinanes, pro-apoptotic hemiacetialic cyclomyrsinanes from *Euphorbia kopetdaghi*. Fitoterapia.

[50] Wongprayoon P., Leelasart S., Jantham J., Pootaeng-on Y., Oekchuae S., Limpachayaporn P., Rayanil K., Charoensuksai P. (2022). A triterpenoid friedelan-3β-ol isolated from *Euphorbia lactea* exhibited cytotoxic activity against HN22 cells by inducing an S-phase cell cycle arrest. J. Appl. Pharm. Sci..

[51] Wang W., Liu Y., Xiong L., Sun D., Wang H., Song Z., Li Y., Li H., Chen L. (2023). Synthesis of Lathyrol PROTACs and evaluation of their anti-inflammatory activities. J. Nat. Prod..

[52] Jiang D., Gao X., Tan R., Liu X., Zhu Y., Zhang L. (2023). *Euphorbia* factor L1 suppresses breast cancer liver metastasis via DDR1-mediated immune infiltration. Aging.

[53] Shi H., Li S., Geng Y., Fan H., Zhang R., Zhang Y., Pan J., Song G., Ge L., Xie T. (2022). *Euphorbia* factor L3 ameliorates rheumatoid arthritis by suppressing the inflammatory response by targeting Rac family small GTPase 1. Bioengineered.

[54] Wang Y., Song Z., Guo Y., Xie H., Zhang Z., Sun D., Li H., Chen L. (2021). Diterpenoids from the seeds of *Euphorbia lathyris* and their anti-inflammatory activity. Bioorg. Chem..

[55] Zuo Q., Mu H.Y., Gong Q., Ding X., Wang W., Zhang H.Y., Zhao W.M. (2021). Diterpenoids from the seeds of *Euphorbia lathyris* and their effects on microglial nitric oxide production. Fitoterapia.

[56] Wang W., Wu Y., Li C., Yang Y., Li X., Li H., Chen L. (2020). Synthesis of new Lathyrane diterpenoid derivatives from *Euphorbia* lathyris and evaluation of their anti-inflammatory activities. Chem. Biodivers..

[57] Wang J.X., Wang Q., Zhen Y.Q., Zhao S.M., Gao F., Zhou X.L. (2018). Cytotoxic Lathyrane-type diterpenes from seeds of *Euphorbia lathyris*. Chem. Pharm. Bull..

[58] Teng Y.N., Wang Y., Hsu P.L., Xin G., Zhang Y., Morris-Natschke S.L., Goto M., Lee K.H. (2018). Mechanism of action of cytotoxic compounds from the seeds of *Euphorbia lathyris*. Phytomedicine.

[59] Wang Q., Zhen Y.Q., Gao F., Huang S., Zhou X. (2018). Five new diterpenoids from the seeds of *Euphorbia lathyris*. Chem. Biodivers..

[60] Lee J.W., Jin Q., Jang H., Kim J.G., Lee D., Kim Y., Hong J.T., Lee M.K., Hwang B.Y. (2018). Lathyrane-type diterpenoids from the seeds of *Euphorbia lathyris* L. with inhibitory effects on NO production in RAW 264.7 cells. Chem. Biodivers..

[61] Xia R.F., Su J.C., Yu J., Zha H.J., Wu J.L., Fu X.N., Cai Q., Wan L.S. (2023). Anti-inflammatory lanostane triterpenoids with rearranged spirobi[indene] scaffold and their biogenetically related analogues from *Euphorbia maculata*. Phytochemistry.

[62] Sun Y., Gao L.L., Tang M.Y., Feng B.M., Pei Y.H., Yasukawa K. (2018). Triterpenoids from *Euphorbia maculata* and their anti-inflammatory effects. Molecules.

[63] Azizi K., Hamedi A., Azarpira N., Hamedi A., Shahini M., Pasdaran A. (2021). A new cytotoxic sesquiterpene lactone from *Euphorbia microsphaera* Boiss against human breast cancer (MCF-7) and human fibrosarcoma (HT1080) cells. Toxicon.

[64] Chang S.S., Huang H.T., Wei W.C., Lo I.W., Lin Y.C., Chao C., Liao G.Y., Shen Y.C., Chen J.J., Li T.L. (2023). Anti-inflammatory effect of euphane- and tirucallane-type triterpenes isolated from the traditional herb *Euphorbia neriifolia* L.. Front. Chem..

[65] Chang S.S., Huang H.T., Lin Y.C., Chao C.H., Liao G.Y., Lin Z.H., Huang H.C., Chun-Ling Kuo J., Liaw C.C., Tai C.J. (2022). Neritriterpenols A-G, euphane and tirucallane triterpenes from *Euphorbia neriifolia* L. and their bioactivity. Phytochemistry.

[66] Yuan G., Jun-Su Z., Hong-Chun L., Yan Z., Wei-Hang Y., Qun-Fang L., Guan-Wu W., Jin-Xin Z., Jian-Min Y. (2022). Phonerilins A–K, cytotoxic ingenane and ingol diterpenoids from *Euphorbia neriifolia*. Tetrahedron.

[67] Li J.C., Feng X.Y., Liu D., Zhang Z.J., Chen X.Q., Li R.T., Li H.M. (2019). Diterpenoids from *Euphorbia neriifolia* and their related anti-HIV and cytotoxic activity. Chem. Biodivers..

[68] Ferreira R.J., Kincses A., Gajdács M., Spengler G., Dos Santos D.J.V.A., Molnár J., Ferreira M.U. (2018). Terpenoids from *Euphorbia pedroi* as multidrug-resistance reversers. J. Nat. Prod..

[69] Chen Y.Y., Zeng X.T., Xu D.Q., Yue S.J., Fu R.J., Yang X., Liu Z.X., Tang Y.P. (2022). Pimarane, abietane, and labdane diterpenoids from *Euphorbia pekinensis* Rupr. and their anti-tumor activities. Phytochemistry.

[70] Li Y.Y., Yang Y., Sun M., Lu Q.Y., Pu X.X., Ran X., Li D.M., Wan J.J., Huang J.Y., Guan S.P. (2022). Jatrophane polyesters from the leaves of *Euphorbia peplus* with anti-inflammatory activity. Phytochem. Lett..

[71] Aljohani A.S.M., Alhumaydhi F.A., Rauf A., Hamad E.M., Rashid U. (2022). In Vivo anti-inflammatory, analgesic, sedative, muscle relaxant activities and molecular docking analysis of phytochemicals from *Euphorbia pulcherrima*. Evid-Based Complement. Altern. Med..

[72] Li M.M., Qi Y.R., Feng Y.P., Liu W., Yuan T. (2022). Euphatexols C - G, five new triterpenoids from the latex of *Euphorbia resinifera*. J. Asian Nat. Prod. Res..

[73] Fantoukh O.I., Al-Hamoud G.A., Nasr F.A., Almarfadi O.M., Hawwal M.F., Ali Z., Alobaid W.A., Binawad A., Alrashidi M., Alasmari F. (2023). Revisiting the flora of Saudi Arabia: Phytochemical and biological investigation of the endangered plant species *Euphorbia saudiarabica*. Metabolites.

[74] Salha M.A., Khaled M., Samir A.M., Abdulrahman I.A., Kamel H.S. (2021). Bioactive compounds from *Euphorbia schimperiana* with cytotoxic and antibacterial activities. S. Afr. J. Bot..

[75] Yang H., Mamatjan A., Tang D., Aisa H.A. (2021). Jatrophane diterpenoids as multidrug resistance modulators from *Euphorbia sororia*. Bioorg. Chem..

[76] Zhu H., Ren X., Huang Y., Su T., Yang L. (2023). Chemical constituents of *Euphorbia stracheyi* Boiss (Euphorbiaceae). Metabolites.

[77] Liu J.L., Yu M., Liao H.B., Liu T., Tan Y.H., Liang D., Zhang G.J. (2019). Sesquiterpenes and diterpenes from *Euphorbia thymifolia*. Fitoterapia.

[78] Duong T.H., Beniddir M.A., Genta-Jouve G., Nguyen H.H., Nguyen D.P., Nguyen T.A., Mac D.H., Boustie J., Nguyen K.P., Chavasiri W. (2019). Further terpenoids from *Euphorbia tirucalli*. Fitoterapia.

[79] Silva V.A.O., Rosa M.N., Miranda-Gonçalves V., Costa A.M., Tansini A., Evangelista A.F., Martinho O., Carloni A.C., Jones C., Lima J.P. (2019). Euphol, a tetracyclic triterpene, from *Euphorbia tirucalli* induces autophagy and sensitizes temozolomide cytotoxicity on glioblastoma cells. Investig. New Drug.

[80] Silva V.A.O., Rosa M.N., Tansini A., Oliveira R.J.S., Martinho O., Lima J., Pianowski L.F., Reis R.M. (2018). In vitro screening of cytotoxic activity of euphol from *Euphorbia tirucalli* on a large panel of human cancer-derived cell lines. Exp. Ther. Med..

[81] Cruz L.S., de Oliveira T.L., Kanunfre C.C., Paludo K.S., Minozzo B.R., Prestes A.P., Wang M., Fernandes D., Santos F.A.D., Manda V.K. (2018). Pharmacokinetics and cytotoxic study of euphol from *Euphorbia umbellata* (Bruyns) Pax latex. Phytomedicine.

[82] Wang Y., Sun D., Jiang Q., Xiong L., Zhang N., Pan Y., Li H., Chen L. (2023). Diterpenoids with anti-inflammatory activity from *Euphorbia wallichii*. Phytochemistry.

[83] Wang Y., Jiang Q., Sun D., Zhang N., Lin Y., Li H., Chen L. (2023). Ent-kauranes and ent-atisanes from *Euphorbia wallichii* and their anti-inflammatory activity. Phytochemistry.

[84] WHO https://www.who.int/news-room/fact-sheets/detail/noncommunicable-diseases.

[85] Benjamaa R., Moujanni A., Kaushik N., Choi E.H., Essamadi A.K., Kaushik N.K. (2022). *Euphorbia* species latex: A comprehensive review on phytochemistry and biological activities. Front. Plant Sci..

[86] Vasas A., Hohmann J. (2014). *Euphorbia* Diterpenes: Isolation, Structure, Biological Activity, and Synthesis (2008–2012). Chem. Rev..

[87] Appendino G. (2016). Ingenane Diterpenoids. Prog. Chem. Org. Nat. Prod..

[88] Zhang Y., Fan R.Z., Sang J., Tian Y.J., Chen J.Q., Tang G.H., Yin S. (2020). Ingol diterpenoids as P-glycoprotein-dependent multidrug resistance (MDR) reversal agents from *Euphorbia marginata*. Bioorg. Chem..

[89] Zhang F., Ma C., Che Q., Zhu T., Zhang G., Li D. (2023). Extending the Structural Diversity of Labdane Diterpenoids from Marine-Derived Fungus *Talaromyces* sp. HDN151403 Using Heterologous Expression. Mar. Drugs.

[90] Yoshinaga K., Yokoshima S. (2023). Convergent synthesis of the [5-7-6-3] tetracyclic core of premyrsinane diterpenes. Org. Biomol. Chem..

[91] Vela F., Ezzanad A., Hunter A.C., Macías-Sánchez A.J., Hernández-Galán R. (2022). Pharmacological Potential of Lathyrane-Type Diterpenoids from Phytochemical Sources. Pharmaceuticals.

[92] Iwata M., Inoue T., Asai Y., Hori K., Fujiwara M., Matsuo S., Tsuchida W., Suzuki S. (2020). The protective role of localized nitric oxide production during inflammation may be mediated by the heme oxygenase-1/carbon monoxide pathway. Biochem. Biophys. Rep..

[93] Greten F.R., Grivennikov S.I. (2019). Inflammation and cancer: Triggers, mechanisms, and consequences. Immunity.

[94] Lin M., Tang S., Zhang C., Chen H., Huang W., Liu Y., Zhang J. (2017). Euphorbia factor L2 induces apoptosis in A549 cells through the mitochondrial pathway. Acta Pharm. Sin. B.

